# Computational Understanding of the Selectivities in Metalloenzymes

**DOI:** 10.3389/fchem.2018.00638

**Published:** 2018-12-21

**Authors:** Wen-Jie Wei, Hui-Xia Qian, Wen-Juan Wang, Rong-Zhen Liao

**Affiliations:** Key Laboratory of Material Chemistry for Energy Conversion and Storage, Ministry of Education, Hubei Key Laboratory of Bioinorganic Chemistry and Materia Medica, Hubei Key Laboratory of Materials Chemistry and Service Failure, School of Chemistry and Chemical Engineering, Huazhong University of Science and Technology, Wuhan, China

**Keywords:** QM, QM/MM, metalloenzyme, selectivity, reaction mechanism

## Abstract

Metalloenzymes catalyze many different types of biological reactions with high efficiency and remarkable selectivity. The quantum chemical cluster approach and the combined quantum mechanics/molecular mechanics methods have proven very successful in the elucidation of the reaction mechanism and rationalization of selectivities in enzymes. In this review, recent progress in the computational understanding of various selectivities including chemoselectivity, regioselectivity, and stereoselectivity, in metalloenzymes, is discussed.

## Introduction

Enzymes play a vital role in many biological processes, such as cell growth, food metabolism, signaling, regulation, energy transduction, and genetic translation (Bugg, 2001). More than 6,000 biochemical reactions have been found to be catalyzed by enzymes, (Schomburg et al., 2017) about one-third of which are metalloenzymes (Ragsdale, 2006). A particular advantage of using enzymes for biotransformation's, is their ability of accelerating chemical reactions by 7 to 19 orders of magnitude in a mild reaction condition (Wolfenden and Snider, 2001). In addition, the enzyme catalysis is generally associated with exquisite selectivity, including chemoselectivity, regioselectivity, and stereoselectivity. However, some enzymes also accept unnatural substrates and show promiscuity, (Khersonsky and Tawfik, 2010), which has been proposed as directly connected to enzyme evolution (Jensen, 1976). An enzyme can also be engineered to accept the non-natural substrate with high efficiency and selectivity. For example, Kan et al. have applied the directed evolution methodology to expand the catalytic function of the cytochrome c, which elegantly mediates the C-Si bond formation with a broad substrate scope as well as with high chemoselectivity and enantioselectivity (Kan et al., 2016).

Understanding reaction mechanisms and selectivities in enzymes, is of fundamental and practical importance. Various experimental techniques have been developed to elucidate many aspects of these two important questions, including X-ray structure analysis, NMR, kinetic analysis, site-directed mutagenesis, isotope labeling, and spectroscopic methods. With the continuous advancement of computer power, the computational chemistry methods have been developed as a crucial complimentary method, to the current experimental methods in the study of enzyme catalysis (Martí et al., 2004; Bruice, 2006; Gao et al., 2006; Warshel et al., 2006; Dal Peraro et al., 2007; Senn and Thiel, 2007a, 2009; Ramos and Fernandes, 2008; Lonsdale et al., 2010, 2012; Siegbahn and Himo, 2011; Rovira, 2013; Blomberg et al., 2014; Merz, 2014; Swiderek et al., 2014; Brunk and Rothlisberger, 2015; Quesne et al., 2016; Sousa et al., 2017; Ahmadi et al., 2018; Cerqueira et al., 2018). From theoretical calculations, different reaction pathways can be analyzed and the structures of transition states and intermediates can be obtained, which are very difficult when using experimental tools. A wealth of new insights can be gained, in particular, selectivities and other important mechanistic aspects, such as kinetic isotope effects (Gao and Truhlar, 2002; Truhlar et al., 2002; Hammes-Schiffer, 2006; Pu et al., 2006), nuclear tunneling (Gao and Truhlar, 2002; Truhlar et al., 2002; Hammes-Schiffer, 2006; Pu et al., 2006) and dynamic effects (Villa and Warshel, 2001; Gao and Truhlar, 2002; Truhlar et al., 2002; Antoniou et al., 2006; Hammes-Schiffer, 2006; Olsson et al., 2006; Pu et al., 2006), can be rationalized.

In general, there are two popular approaches in the modeling of enzymes. The first one is called the quantum chemical cluster method, developed by Siegbahn, Blomberg, Himo, et al. (Siegbahn and Blomberg, 1999, 2000; Himo and Siegbahn, 2003; Noodleman et al., 2004; Siegbahn and Borowski, 2006; Siegbahn and Himo, 2009, 2011; Blomberg et al., 2014; Himo, 2017). In this approach, with more than 20 years' experience, the small model of the active site is capable of capturing the key feature of the catalysis. At the beginning, cluster models of 30–50 atoms were commonly used and nowadays cluster models of more than 200 atoms are routinely handled. The second approach is the combined quantum mechanics/molecular mechanics (QM/MM) method, which was first proposed by Warshel and Levitt (1976). In this case, the whole solvated enzyme was typically used as the model. Two QM/MM protocols were commonly applied, namely electronic energy calculations from geometry optimization starting from the X-ray structure or certain snapshots from the trajectory of a classical molecular dynamics simulations, and free energy calculations from QM/MM molecular dynamics simulations (Martí et al., 2004; Bruice, 2006; Gao et al., 2006; Warshel et al., 2006; Dal Peraro et al., 2007; Senn and Thiel, 2007a, 2009; Ramos and Fernandes, 2008; Lonsdale et al., 2010, 2012; Rovira, 2013; Merz, 2014; Swiderek et al., 2014; Brunk and Rothlisberger, 2015; Quesne et al., 2016; Sousa et al., 2017; Ahmadi et al., 2018; Cerqueira et al., 2018). With a proper selection and an increase of the size of the QM region, Liao, and Thiel have shown that the cluster approach and the QM/MM geometry optimization protocol gave similar results and the same conclusion (Liao and Thiel, 2012, 2013a).

In this review, the main focus was on the selectivities of metalloenzymes, for which the elucidation of the enzymatic mechanism was a perquisite. The reproduction of the selectivity can be considered as further support for the suggested mechanism. It should be pointed out that not all computational studies from the literature can be discussed, therefore, a number of illustrative examples have been chosen for each type of metalloenzymes, covering Mn, Fe, Co, Ni, Zn, Mo, and W. Recently, de Visser presented an excellent summary of the substrate selectivity of non-heme iron dioxygenases (de Visser, 2018).

## Methods and Models

In the quantum chemical cluster modeling of metalloenzymes, a model of the active site is designed, typically containing the metal ions with their first-shell ligands and some important second-shell ligands. The whole model can be treated at the highest possible level, mainly hybrid density functional methods with very large basis sets.

The protein environment has two major effects, sterically and electrostatically, which can be covered in a simple but effective manner. The steric effects imposed by the protein matrix are taken into account using the coordinate-locking scheme, in which certain atoms are fixed at their X-ray structure positions. The general experience is to select those atoms at the periphery of the model, typically the α-carbon atoms of the residues where the truncation is introduced. In some cases, one or two more hydrogen atoms attached to each α-carbon atom along the backbone are fixed to avoid unrealistic rotation of the residues. Due to the usage of the coordinate-locking procedure, the accuracy of the results from the cluster calculations may depend on the quality (or the coordinate error) of the crystal structure used. With acetylene hydratase as an example, Liao and Thiel have demonstrated that a coordinate error of 0.1 Å for the backbone atoms, corresponding to a resolution of about 2.0 Å for the crystal structures, result in tolerable errors (about 1 kcal/mol for relative energies and 0.01 Å for bond distances) for cluster models with about 100 atoms (Liao and Thiel, 2013b). Larger models with more flexibility may be needed to obtain reliable results when starting from a crystal structure with a lower resolution and larger coordinate error. For residues that participate directly in the catalyzed reaction, it is advisable to lock atoms further away than the α-carbon.

The electrostatic interactions between the model and the surroundings, are treated using continuum solvation model methods, such as the SMD solvation method (Marenich et al., 2009), typically with a dielectric constant of 4 (Blomberg et al., 2014). For most enzymatic reactions, in which the total charge of the model does not change during the reaction, the choice of the dielectric constant has been shown to be unimportant when the cluster model reaches a size of around 200 atoms. This kind of phenomenon has been observed for four different electrostatically challenging examples, namely 4-oxalocrotonate tautomerase (Sevastik and Himo, 2007), haloalcohol dehalogenase (Hopmann and Himo, 2008), histone lysine methyltransferase (Georgieva and Himo, 2010), and aspartate decarboxylase (Liao et al., 2011a). However, when the total charge of the model changes, such as in the calculations of an electron redox potential and pKa values, the solvation effects become very large and also sensitive to the choice of the dielectric constant (Liao et al., 2015, 2016). An error of 5–10 kcal/mol may be expected, which is too large to be ignored. It should be pointed out that an empirical method has been developed by Siegbahn in the study of the oxygen evolving complex in photosystem II, for which a single parameter, derived from fitting the available experimental data, was used to determine the redox potentials and pKas (Siegbahn, 2013). This approach has also been successfully applied in the study of Cytochrome c Oxidase (Blomberg and Siegbahn, 2010), nitric oxide reductase (Blomberg and Siegbahn, 2013), and nitrogenase (Siegbahn, 2017).

The most popular functional applied for metalloenzymes is the B3LYP (Becke, 1993) functional (with 20% HF exchange), for which the major improvement is the addition of the empirical D3 dispersion corrections as proposed by Grimme (termed B3LYP-D3) (Grimme et al., 2010). Other functionals, such as B3LYP^*^-D3 (with 15% HF exchange; Reiher et al., 2001), TPSSh-D3 (Staroverov et al., 2003), and the M06 series (Zhao and Truhlar, 2008), can be applied to examine the sensitivity of the results to the choice of functional. Geometry optimizations are usually carried out by using a double-zeta quality basis set with effective pseudopotentials on the metals, while all electron basis sets may also be used. The final energies are then estimated by performing single-point calculations using larger basis sets. Analytic harmonic frequencies are then computed at the same level of theory as the geometry optimizations. Due to the coordinate-locking scheme used, a number of small imaginary frequencies are obtained, which makes the entropy effects difficult to predict. Therefore, only the zero-point energy corrections are included in the final energies.

In the alternative QM/MM methodology, the protein surrounding, with the water solvent and counter-ions are treated at the classical molecular mechanics level. Depending how the coupling between the QM and the MM part is performed, the method can be divided into two classes, additive methods and subtractive methods. In the additive QM/MM method, the total energy of the system is calculated as the sum of the individual energies of the QM and MM parts (two independent calculations), plus a QM/MM coupling term (Equation 1):

(1) $$\begin{array}{l} {\text{E}}_{\text{QM}/\text{MM},\text{fullsystem}}={\text{E}}_{\text{QM},\text{innerlayer}}+{\text{E}}_{\text{MM,outerlayer}}\\ \;+{\text{E}}_{\text{QM}/\text{MM},\text{coupling}} \end{array}$$

In the subtractive QM/MM method, different parts are subjected to independent calculations at different levels of theory, and the total energy of the system is then obtained by additions and subtractions. With a typical two-layered system as an example, the energy of the whole system is calculated at the MM level, followed by energy calculations of the inner layer at both the QM level and the MM level. The final total energy is then expressed as Equation 2:

The “our own n-layered integrated MO and MM” method (ONIOM) developed by Morokuma and co-workers is nowadays a very popular subtractive QM/MM method. (Chung et al., 2012).

The boundary problem, which occurs when covalent bonds are presented between the QM and MM parts, can be handled using two common strategies, namely, the frozen orbital approach (Thery et al., 1994) and the link atom (typically hydrogen) approach (Singh and Kollman, 1986).

(2) $${\text{E}}_{\text{QM}/\text{MM},\text{fullsystem}}={\text{E}}_{\text{MM,fullsystem}}-{\text{E}}_{\text{MM},\text{innerlayer}}+{\text{E}}_{\text{QM},\text{innerlayer}}$$

The interactions between the QM and MM regions are approximately categorized into two parts, namely, van der Waals interactions and electrostatic interactions. The van der Waals interactions are usually described by an empirical Lennard–Jones potential. The electrostatic interactions can be treated at three different levels of sophistication, namely, mechanical embedding, electrostatic embedding, and polarized embedding. In the mechanical-embedding scheme, the QM–MM electrostatic interactions are treated as the MM-MM electrostatics at the classical-classical level, in which the electrostatic effect of the MM environment on the QM region is neglected and the QM density is not polarized. In the electrostatic embedding scheme, the MM point charges are incorporated as one-electron terms in the QM Hamiltonian. Therefore, the QM–MM electrostatic interactions are treated at the quantum-classical level, and the QM density is polarized by the MM environment. In the polarized embedding scheme, a flexible MM charge model is introduced, which is polarized by the QM charge distribution. The mutual polarization of both QM and MM regions can be treated using a self-consistent formulation. Electrostatic embedding is still the most popular choice due to its tradeoff between accuracy and efficiency. Due to the artificial truncation of the QM and MM regions, the potential charge transfer between these two regions cannot be handled using these schemes in the QM/MM calculations. A simple and straightforward way to minimize the error of using force fields for the MM region, is to increase the size of the QM region by proper selection and inclusion of residues around the active site, into the QM region. A simple procedure is to select residues using the distance criteria. Alternatively, the charge deletion analysis (Bash et al., 1991) has been suggested to identify those residues that have strong electrostatic effects on the relative energies along the reaction pathways (Liao and Thiel, 2012, 2013a); Two different stationary points with the largest charge relocalization can be selected for single-point calculations, in which the point charges of every residue close to the active site are set to zero. Special attention should be given to residues or groups with more than one positive/negative charge, for example, a diphosphate group (Liao and Thiel, 2012). The magnitude of the change of the relative energy, upon the removal of the MM point charges of the selected residue, can be used to quantify the electrostatic contribution of each residue. The general idea is that if the residue gives quite a large relative energy difference in the analysis, then this residue should be selected into the QM region to get reliable results (Liao and Thiel, 2012, 2013a); By using this protocol, one can systematically increase the size of the QM region to check the convergence behavior of the QM/MM energies. Various different properties of enzymes and proteins have been investigated with respect to the enlargement of the QM size, for example, NMR shielding (Flaig et al., 2012; Hartman et al., 2015), excitation energies (Isborn et al., 2012; Provorse et al., 2016; Milanese et al., 2017), barrier heights (Solt et al., 2009; Liao and Thiel, 2013a; Sadeghian et al., 2014; Jindal and Warshel, 2016; Kulik et al., 2016; Karelina and Kulik, 2017; Das et al., 2018); reaction energies (Fox et al., 2011; Hu et al., 2011, 2013; Roßbach and Ochsenfeld, 2017), and others (Karelina and Kulik, 2017; Morgenstern et al., 2017).

Two different strategies have been developed in the QM/MM calculations. One is to perform QM/MM geometry optimizations using the X-ray structure directly (single-conformation) or using selected snapshots (multi potential energy surfaces) from a classical molecular dynamics trajectory. In this case, the QM/MM calculations must ensure that all stationary points are in the same local minima along each reaction path. To solve the conformational complexity problem and also to speed up the QM/MM calculations, an active region of about 1,000–1,500 atoms around the QM region can be selected while the outer part remains fixed during the geometry optimizations (Shaik et al., 2010). When a large conformational change is presented during the reaction, especially for substrate binding and product release, the QM/MM molecular dynamics and Monte Carlo simulations can be used, along with the standard free-energy methods, such as free-energy perturbation, umbrella sampling, and thermodynamic integration (Senn and Thiel, 2007b). The QM/MM free energy calculations, which often require sufficiently long simulation time scales to achieve convergence, are much more time-demanding than the QM/MM geometry optimization calculations. As a compromise, a relatively smaller QM region and cheaper method, such as the semi-empirical methods and DFT functionals with double-zeta quality basis sets, are commonly used. Importantly, Senn et al. have shown that the differences between QM/MM electronic energy and free energy profiles are quite small in the study of local chemical events (Senn et al., 2009).

When performing QM/MM calculations on enzymes, a considerable amount of work has to be invested into the setup of the system before the actual QM/MM calculations. First, MM parameters are needed for the whole system. These parameters may not be available for substrates, cofactors and metals, which could be restrained to their X-ray positions in order to avoid the need to develop bonded parameters. However, non-bonded parameters, in particular atomic charges, should be developed at a reasonable level. The point charges are commonly derived from QM calculations on the selected molecules. The starting X-ray structure needs to be checked and revised prior to classical molecular mechanics simulations. Missing hydrogen atoms should be added, the protonation states of all titrable residues (e.g., His, Asp, and Glu) should be assigned according to their pKa values and local hydrogen bonding networks, the orientations of amide groups of Asn and Gln, as well as imidazole group of His, should be checked and flipped if needed. For the calculations of pKas, the empirical algorithms of PROPKA (Olsson et al., 2011) is a suitable choice, but a more rigorous solution can be obtained using Poisson-Boltzmann or QM/MM methods. The enzyme is then solvated into a water box under periodic boundary conditions or a spherical water droplet with a sufficiently large radius under spherical boundary potential. In the following step, the whole system is neutralized by either adding counter ions or by (de)protonation of charged residues at the surface of the enzyme. After these initial preparations, constrained energy minimizations, and classical molecular dynamics simulations can be performed, from which a number of snapshots can be selected as the starting geometries for the following QM/MM calculations. In the QM/MM calculations, the choice of QM method is essentially the same as that in the cluster approach, and the standard biomolecular force fields, such as Charmm (MacKerell et al., 1998), Amber (Duan et al., 2003), and Gromos (Schmid et al., 2011) are often used for the MM treatment. With the development of the very efficient domain-based local pair natural orbital coupled cluster method with single, double, and perturbatively included triple excitations (DLPNO-CCSD(T)) from the Neese group (Neese et al., 2009), it is advisable to perform QM/MM single-point calculations using DLPNO-CCSD(T) with large basis sets to obtain more accurate energies (Bistoni et al., 2018).

Various sources of errors can be envisioned from the QM/MM calculations, the setup and the starting conformation of the model system, the choice of the QM region, the choice of the QM method and MM method, the treatment of the QM/MM boundary, the entropy effects, and so on. Due to the different choices of QM/MM calculations, it is likely that different research groups obtain different results, but in general the same conclusion.

For the investigation of selectivities in enzymes, an important question is to uncover the origin of the selectivity. For metalloenzymes, both the metals and the active site residues could be involved in controlling the selectivity. A number of analytic tools from calculations could be used to find the factors that dictate the selectivity, such as frontier molecular orbitals, Fukui functions, distortion/interaction analysis (Ess and Houk, 2008; Fernández and Bickelhaupt, 2014; Bickelhaupt and Houk, 2017), and residue contribution analysis (Bash et al., 1991; Karelina and Kulik, 2017).

## Modeling Selectivities in Metalloenzymes

### Mn-Dependent Enzymes

Two different groups have investigated two different Mn-dependent enzymes that showed selectivities. One is a non-redox enzyme FosA with regioselectivity and chemoselectivity (Rife et al., 2002), and the other is a redox-active enzyme QueD with regioselectivity (Gopal et al., 2005).

**Figure 1:**
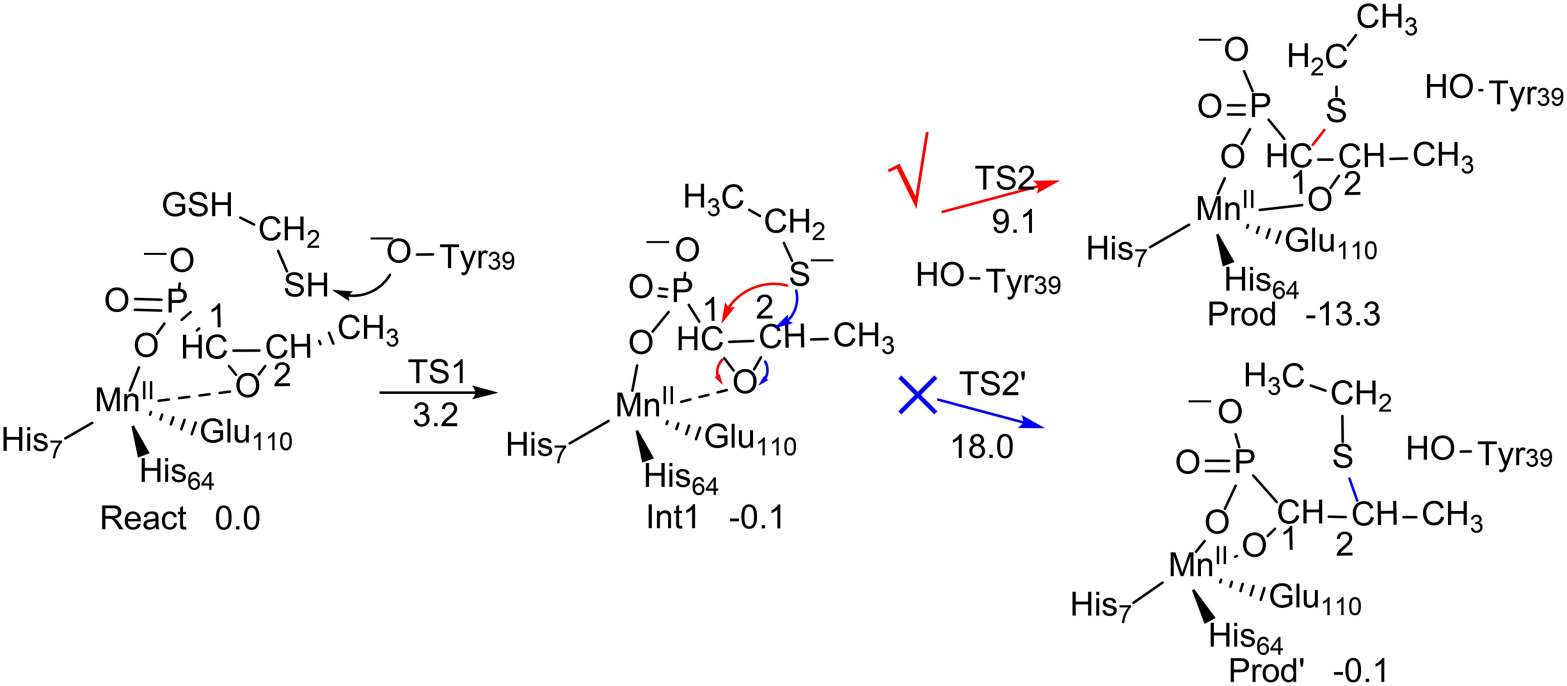
Reaction pathways considered for FosA. QM/MM (B3LYP/def2-TZVPP:Charmm) relative energies are given in kcal/mol (Liao and Thiel, 2013c).

FosA (Rife et al., 2002) is a manganese-dependent Fosfomycin resistance protein that catalyzes the inactivation of the Fosfomycin antibiotic by the nucleophilic addition of glutathione (GSH) on the epoxide ring (Figure 1) with high regioselectivity, in which the attack exclusively takes place at the C1 position. Interestingly, the uncatalyzed reaction in water solution was shown to be essentially unselective (yield of 40% on C1 and 60% on C2) (Bernat et al., 1999). There is another Fosfomycin resistance protein named FosX that mediates a nucleophilic water attack on the epoxide ring (Fillgrove et al., 2003). This also raises the question as to why FosA does not catalyze the addition of a water molecule on Fosfomycin, even though the enzyme has an open active site pocket for water binding. Consequently, FosA is both regioselectivity and chemoselective (Bernat et al., 1998).

Liao and Thiel have performed QM/MM calculations with quite a large QM region of 170 atoms, to elucidate the reaction mechanism and selectivities of FosA (Liao and Thiel, 2013c). The uncatalyzed reaction was first considered using a model consisting of a Fosfomycin and methanethiol as a model substrate for GSH, and two water molecules. Importantly, the calculations showed that the attacks on both C1 and C2 take place in a concerted step with a very similar barrier, of around 30 kcal/mol, which explains the experimental observation for the uncatalyzed reaction (Bernat et al., 1999). In FosA, the reaction starts from a proton transfer from the GSH thiol group to a second-shell anion residue Tyr39, which is followed by the nucleophilic attack of the GSH thiolate on the epoxide, leading to the opening of the epoxide ring. The second step was suggested to be the rate-limiting step, with a barrier of 9.1 kcal/mol for the attack on C1, while it was 18.0 kcal/mol for the attack on C2. A distortion/interaction analysis (Ess and Houk, 2008; Fernández and Bickelhaupt, 2014; Bickelhaupt and Houk, 2017) was applied to understand the origin of the regioselectivity. It was shown that the distortion energy for **TS2**_**C2**_ (27.1 kcal/mol) is much larger than that for **TS2**_**C1**_ (20.0 kcal/mol), while the interaction energies were quite similar (−16.7 kcal/mol for **TS2**_**C2**_vs. −14.4 kcal/mol for **TS2**_**C1**_). These results suggested that the regioselectivity for the GSH attack is mainly distortion-controlled.

To understand the chemoselectivity, the water attack pathway was also taken into consideration. Different from the GSH attack, the deprotonation of the water substrate, the nucleophilic attack, and the ring-opening of the epoxide proceed in a single concerted step. The energy barrier was found to be 8.3 kcal/mol higher than that of the GSH attack. In addition, the attack on C2 is now preferred. However, the calculated barrier of 17.2 kcal/mol seems to be underestimated.

**Figure 2:**
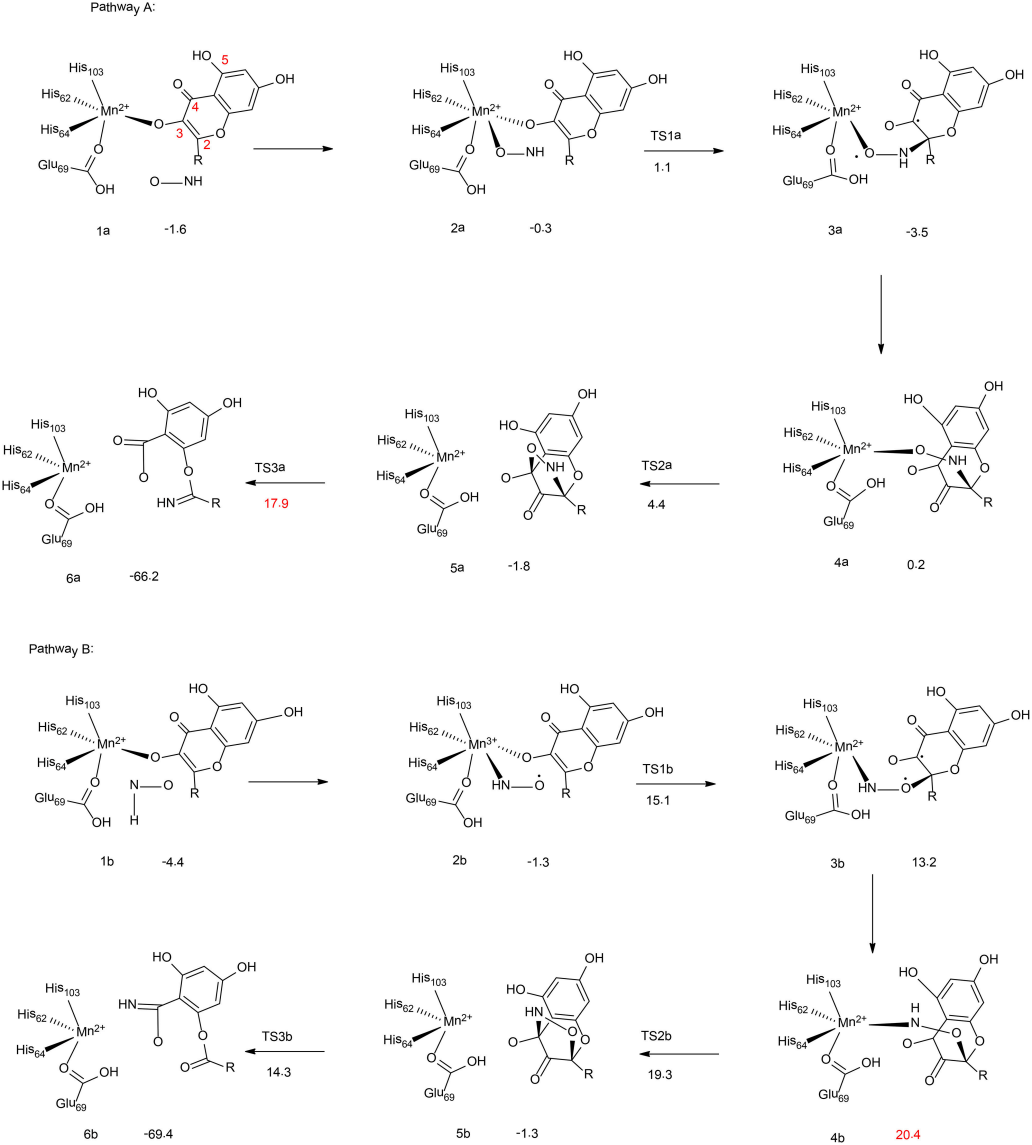
Reaction pathways considered for Mn-QDO. Relative energies are given in kcal/mol relative to the separated complex. (B3LYP-D3/6-311G (d,p)) (Wojdyła and Borowski, 2016). Copyright 2016 Springer Nature.

Quercetin 2,3-dioxygenase (QDO) from *Bacillus subtilis* is a metalloenzyme *that* is capable of using different transition metal ions (Mn2+, Co2+, Fe2+, and Cu2+) to catalyze the dioxygenation of quercetin to generate 2-protocatechuoylphloroglucinol carboxylic acid and carbon monoxide (Gopal et al., 2005). Interestingly, the Mn-QDO shows a nitroxygenase activity in the presence of HNO with high regioselectivity, and the sole product is 2-((3,4-dihydroxyphenyl)(imino)methoxy)-4,6-dihydroxybenzoate (Figure 2). However, the Fe-QDO and the Co-QDO cannot catalyze the nitroxygenation reaction, implying a unique reactivity of the Mn-QDO (Kumar et al., 2011).

Wojdyla and Borowski have performed quantum chemical cluster calculations to elucidate the reactivity and regiospecificity of the Mn-QDO (Wojdyła and Borowski, 2016). The uncatalyzed reaction was first considered with a model containing a quercetin anion and a HNO molecule. The reaction started with the formation of a van der Waals complex. Then, both the nitrogen atom (pathway I) and the oxygen atom (pathway II) of the HNO molecule could perform an electrophilic attack on the C2 atom of the quercetin anion. This is followed by the formation of a five-membered ring intermediate, from which the ring cleavage takes place, coupled with the release of a CO molecule. The calculation showed that in pathway I the final step is rate-limiting step, associated with a barrier of 14.7 kcal/mol. While for pathway II, the rate-limiting step is the five-membered ring formation, with a barrier of 22.6 kcal/mol. Additionally, the Fukui function has been used to analyze the regioselectivity, and pathway I is more favored.

In the Mn-QDO enzyme, the HNO molecule could bind to the Mn^2+^ ion either via the oxygen atom (2a, Figure 2, pathway A) or the nitrogen atom (2b, Figure 2, pathway B). In pathway A, the nitrogen atom attacks the C2 atom of quercetin, (2a → 3a), which is followed by a conformation change (3a → 4a) and the five-membered ring formation (4a → 5a). The last step is the cleavage of the five-membered ring (5a → 6a), which is the rate-limiting step for the whole reaction, with a barrier of 17.9 kcal/mol. For pathway B, the barrier for the first C2-O bond formation is more than 10 kcal/mol higher than that of the first C2-N bond formation. In addition, the rate-limiting step was found to be the hydrogen bond shift process (3b → 4b), with a barrier of 20.4 kcal/mol. The total barrier for pathway B is thus 2.5 kcal/mol higher than that of pathway A, which explains the regioselectivity in the Mn-QDO catalyzed nitroxygenation reaction.

To understand the metal selectivity, the substitution of Mn^2+^ by Co^2+^ and Fe^2+^ in the enzyme active site has also been taken into consideration. The calculations indicated that for both Co-QDO and Fe-QDO, the HNO prefered to be coordinated to the metal via its nitrogen atom. For both cases, the energies of the oxygen-coordinated complexes were more than 6 kcal/mol higher than those of the nitrogen-coordinated complexes. The strong preference for N-coordination over O-coordination explains that the Co-QDO and the Fe-QDO cannot catalyze the nitroxygenation reaction.

### Heme-Dependent Enzymes

**Figure 3:**
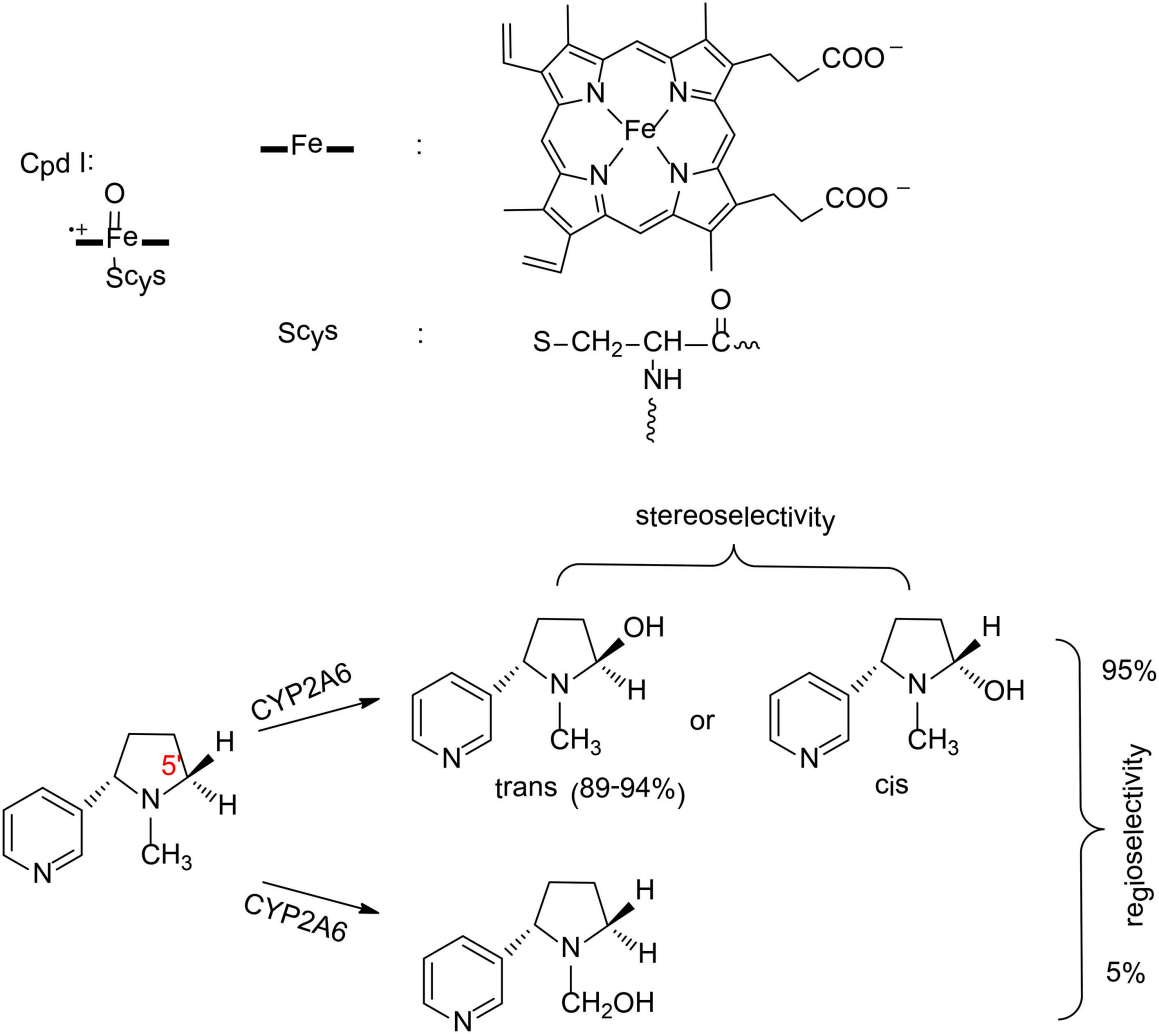
The structure of compound I and reaction catalyzed by P450 2A6 (Li et al., 2011, 2013). Copyright 2013 Royal Society of Chemistry.

Cytochrome P450 is a large superfamily of enzymes that are capable of catalyzing many different types of oxidation reactions. In P450, the key part of the active site is a heme group, and the catalytic active species is a high–valent Fe^IV^-oxo complex with a porphyrin radical cation, termed compound I **(Cpd I**, Figure 3). Due to the great importance of P450, extraordinary efforts have been dedicated to mechanistic studies of these enzymes using computational tools (de Visser and Shaik, 2003; Shaik et al., 2010; Li et al., 2011, 2013; Oláh et al., 2011; Krámos et al., 2012; Blomberg et al., 2014; Dubey et al., 2016; Faponle et al., 2016). Some representative examples of selectivities in P450 investigated theoretically are shown here.

P450 2A6 plays an important role in the metabolism of nicotine in humans (Yamazaki et al., 1999). Two principal pathways were observed for the oxidation of nicotine by P450 2A6, namely hydroxylation at C5′ position and at C_α_ position (Figure 3; Jone et al., 1993). Experimentally, 95% of the product was the cotinine (C5′ hydroxylation; Murphy et al., 2005), which suggested a regioselectivity in this enzyme. In addition, C5′ hydroxylation can lead to either a *trans* or *cis* product, and the major product is the *trans*-5'-hydroxynicotine (Peterson et al., 1987), which implies a stereoselectivity in P450 2A6.

Zhan et al. have performed MD simulations and QM/MM calculations to uncover the origin of the regioselectivity and stereoselectivity of the oxidation of nicotine by P450 2A6 (Li et al., 2011, 2013). From MD simulations, six representative substrate binding models can be located. However, only three of them are suitable for the 5'-hydroxylation, which are labeled as CYP2A6-SR_t_, CYP2A6-SR_c_, and CYP2A6-SR_H_. The MD simulations showed that the binding free energies of these three models are −6.86, −5.42, and −2.04 kcal/mol, respectively. When the distribution of the conformation of the free substrate in solution was taken into consideration, the CYP2A6-SR_t_ complex is the major structure (95.4%), while 4.4% for the CYP2A6-SR_c_ complex.

These two complexes were chosen to rationalize the regioselectivity and stereoselectivity by performing QM/MM calculations. The oxidation mechanism is a typical Cpd I mediated hydroxylation, namely C-H activation via hydrogen transfer from the substrate to the oxyl group, followed by a rebound of the hydroxyl to the substrate. For the C5′ oxidation, the first hydrogen abstraction is the rate-limiting step, and the barriers for the C5′-*trans* and the C5′-*cis* hydroxylations are 14.1 and 14.4 kcal/mol, respectively. Very close barriers indicate a competition between these two pathways. Thus, the free energy barrier alone cannot explain the stereoselectivity of P450 2A6. Instead, the distribution of CYP2A6-SR_t_ and CYP2A6-SR_c_ plays an important role in controlling the stereoselectivity. When this factor was considered, 97% of the product was the *trans*-5'hydroxylation product, which is in excellent agreement with the experimental result of 89–94% (Peterson et al., 1987). These results suggested that the stereoselectivity of P450 2A6 is primarily controlled by the substrate binding mode in the active site.

For the N-methylhydroxylation, the reaction mechanism is very similar. The first hydrogen transfer was found to be the rate–limiting step, with a barrier of 15.5 kcal/mol for the CYP2A6-SR_t_ complex and 18.0 kcal/mol for the CYP2A6-SR_c_ complex. On the basis of the conventional transition-state theory, the final phenomenological free energy barriers for the 5'-hydroxylation and the N-methylhydroxylation were estimated to be 14.1 kcal/mol and 15.6 kcal/mol, respectively. Consequently, the 5'-hydroxylation was kinetically more favorable, corresponding to a regioselectivity of 93%. This is very close to the experimental result of 95% (Murphy et al., 2005).

**Figure 4:**
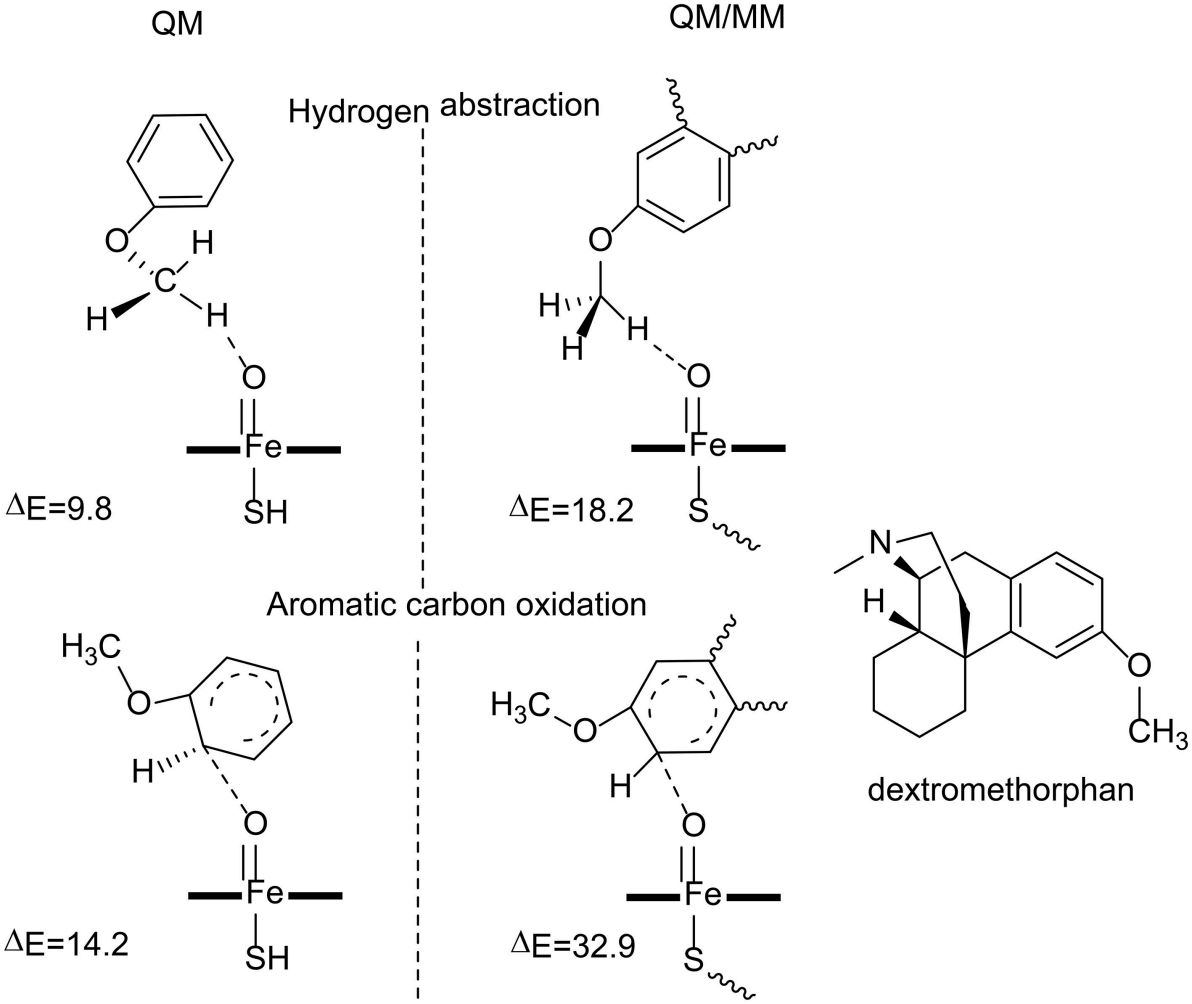
The transition state structures and the barriers (kcal/mol) in the reaction catalyzed by P450 2D6 (Oláh et al., 2011). Copyright (2011) National Academy of Sciences.

P450 2D6 is a human cytochrome P450 enzyme that metabolizes the cough suppressant drug dextromethorphan. Two possible pathways can be envisioned for the oxidation of dextromethorphan, namely O-demethylation and aromatic carbon hydroxylation. (Figure 4) Interestingly, only the former reaction is observed in P450 2D6 catalyzed dextromethorphan oxidation, despite the fact that the aromatic carbon hydroxylation is a major route in the metabolism of other anisole derivatives (Ohi et al., 1992). Therefore, a chemoselectivity is presented in P450 2D6.

Oláh et al. have carried out both QM and QM/MM calculations to understand why the aromatic carbon oxidation route does not occur in the oxidation of dextromethorphan catalyzed by P450 2D6 (Oláh et al., 2011). Both reaction pathways were considered in their study (Figure 4). For the QM calculations, a small model was used, in which the dextromethorphan was represented by an anisole molecule and Cpd I was represented by an un-substituted heme ring with a ferryl oxo and a hydrogen sulfide ligand. In the O-demethylation pathway, the reaction started with a hydrogen atom transfer from the methyl group of the substrate to the oxygen atom of Cpd I, followed by the rebound of the hydroxyl group to the substrate. After the release of the hemiacetal species, a hydrolysis reaction took place with the production of the phenolic product. The first H-abstraction was found to be the rate-limiting step, with a barrier of only 9.8 kcal/mol. In the alternative aromatic carbon oxidation pathway, the oxyl group attacked the aromatic ring, followed by a rearrangement of the tetrahedral intermediate to generate the phenol product. The rate-limiting step was the first C-O bond formation, with a barrier of 14.2 kcal/mol. The QM calculations suggested that both pathways are viable options despite the fact that the O-demethylation route is more preferred.

The QM calculations with a small model does not consider the protein environment in a proper manner, which seems to be very important to rationalize the chemoselectivity. QM/MM calculations were performed using the full enzyme model, and three snapshots were taken from MD simulations and considered. For the O-demethylation route, the energy barriers for the first H-abstraction step for the three snapshots were 18.2, 19.0, and 19.7 kcal/mol, respectively. While for the aromatic carbon oxidation route, the energy barriers for the first C-O bond formation step were 32.9, 33.5, 36.7 kcal/mol, respectively. The high energy barrier for the aromatic carbon oxidation route can safely rule out this pathway, which is in line with the experimental observation (Schmider et al., 1997). Compared with the QM calculations, the QM/MM calculations suggested that the interactions between the dextromethorphan substrate and some key residues (Ser304, Ala305, Val308, and Thr309) in the active site imposed constrains on the movement of the aromatic ring, which increased the energy barrier for the C-O bond formation. The enzyme environment therefore plays an important role in dictating the chemoselectivity of P450 2D6.

**Figure 5:**
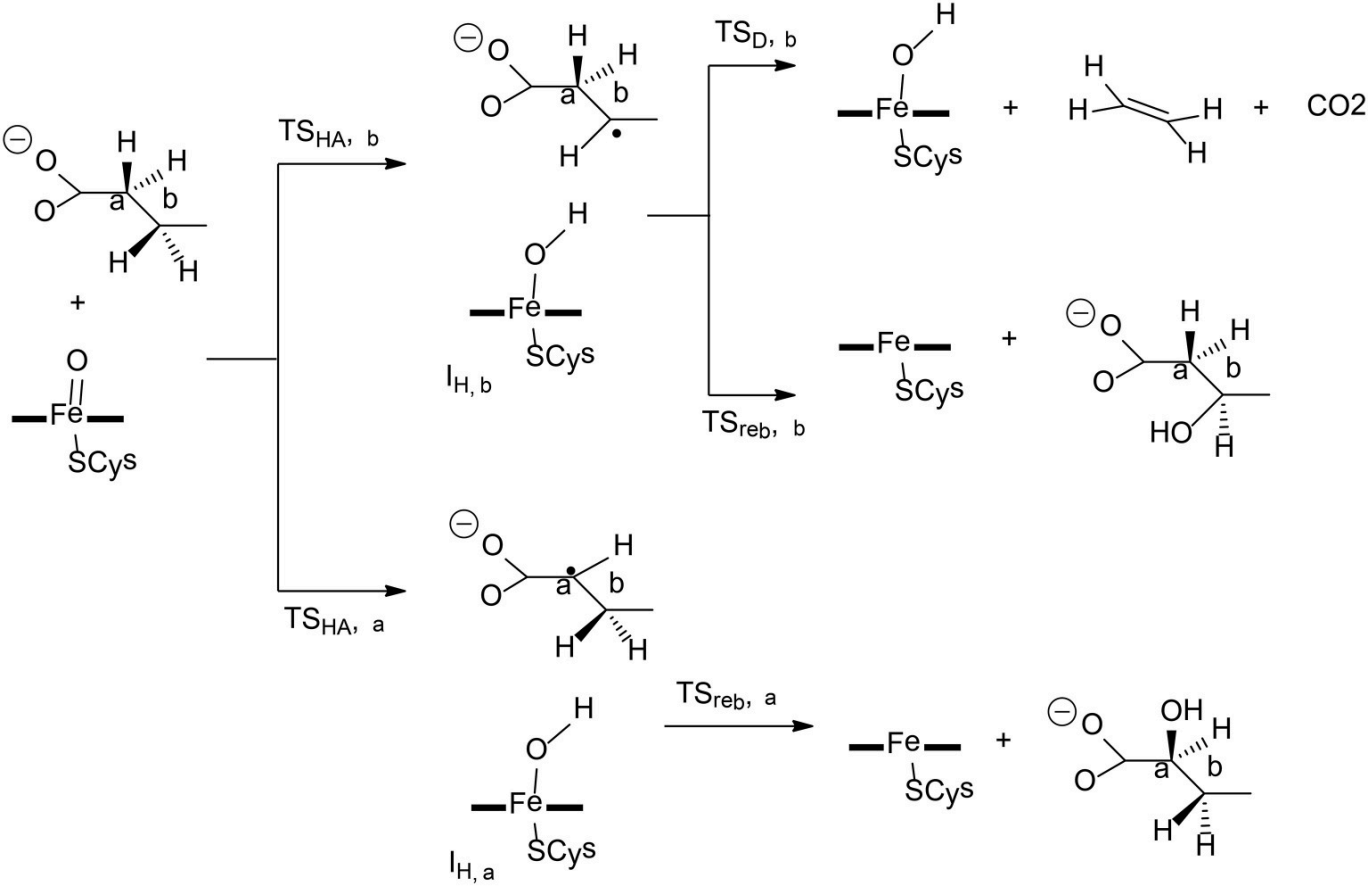
Reaction catalyzed by P450 OleT_JE_ (Faponle et al., 2016). Copyright (2016) John Wiley and Sons.

P450 OleT_JE_ is a peroxygenase catalyzing the conversion of long-chain fatty acids to terminal olefin, which can be used for biofuels (Dennig et al., 2015; Grant et al., 2015). In the oxidation of fatty-acid, three different pathways can be located, namely, α-hydroxylation, β-hydroxylation, and decarboxylation (Figure 5). Faponle et al. have performed QM/MM calculations to understand the regioselectivity and chemoselectivity in this enzyme (Faponle et al., 2016).

The first hydrogen abstraction takes place either at the C_α_ or the C_β_ position of the substrate, and the calculations gave a barrier of 6.4 kcal/mol for TS_HA, α_ and a barrier of 6.3 kcal/mol for TS_HA, β_. During the reaction, a substrate radical and an iron (IV)-hydroxo species are formed, which are labeled as I_H,α_ for α-hydroxylation and I_H,β_ for β-hydroxylation. From I_H,α_, the rebound pathway to form a C-OH bond is very facile, with a barrier of only 7.3 kcal/mol. The decarboxylation from I_H,α_ was found to be associated with a barrier of over 40 kcal/mol, which is too high to be a viable option. However, from I_H,β_, both the rebound process and the decarboxylation reaction can take place, and the corresponding barriers were calculated to be 9.3 and 6.7 kcal/mol, respectively. A valence bond model was used to analyze these two different pathways. It was demonstrated that electron-withdrawing substitutions at the C_α_ or C_β_ position increased the barriers for the rebound process, in turn, making the decarboxylation easier.

**Figure 6:**
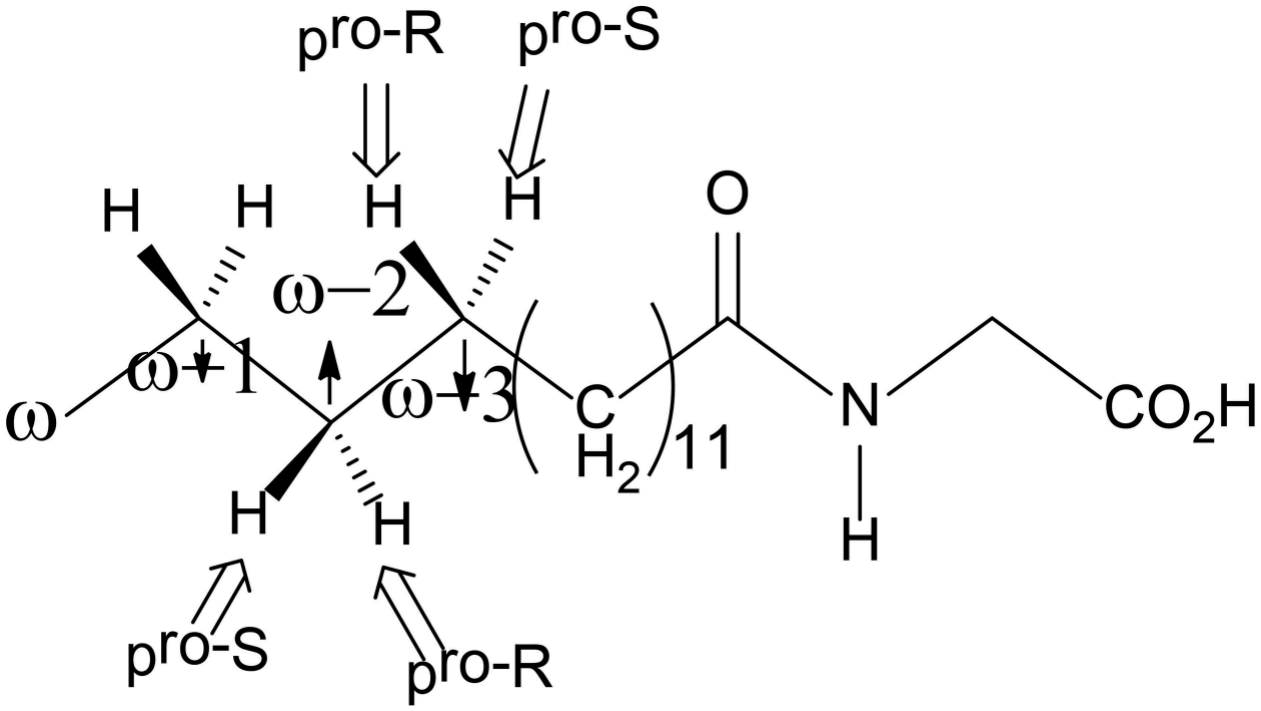
Structure of the NPG substrate.

P450 BM3 catalyzes the C-H hydroxylation of the fatty acid derivative N-palmitoylglycine (NPG) at the ω-1, ω-2, and ω-3 positions of the long chain, but not the terminal ω position, even though the terminal ω-carbon is closer to the heme iron in all of the crystal structures of NPG-bound P450 BM3 (Figure 6; Haines et al., 2001). Shaik et al. have used MD and QM/MM approaches to investigate the regio- and stereo-selectivity in P450 BM3 (Dubey et al., 2016). They first performed MD simulations on three different structures, namely, the open form (the substrate-free enzyme), the closed form (the substrate-bound enzyme), and the enzyme with substrate docked into the open form, to investigate the substrate binding process and to understand how the enzyme is prepared for catalysis. The MD simulations showed that during the binding of the substrate into the active site, the active site undergoes large conformational changes and some key residues play an important role in orientating the substrate to become exposed to the active oxidant.

To explain the regioselectivity, they performed MD simulations for two different states, namely the resting state and the Cpd I state. For the resting state, the calculations showed that in the beginning the ω carbon is the nearest to the heme iron, with a distance of 4–5 Å, while they are in the range of 6 to 8 Å for the ω-1, ω-2, and ω-3 carbons. After 275 ns, the terminal ω carbon becomes about 8 to 12 Å far away from the heme iron. In the case of the Cpd I state, a similar trend was observed. As a consequence, the MD simulations indicated that the favorable positions for C-H hydroxylation are the ω-1/ ω-2 /ω-3 sites, which are in good agreement with the experiment observation (Cryle and De Voss, 2006). They also performed another MD simulation, in which the Phe87 residue was replaced by a smaller Alanine residue. After 200 ns, the terminal ω position becomes the closest to Cpd I, while the ω-1, ω-2, and ω-3 positions become far away from the oxidant. These results are in line with the experiment, in which more than 90% ω hydroxylation product is formed upon Phe87Ala mutation (Dubey et al., 2016). Therefore, Phe87 imposes steric hindrance to the terminal ω-carbon and controls the regioselectivity of P450 BM3.

For the ω-1, ω-2, and ω-3 hydroxylations, the first H-abstraction is the rate-limiting step. In all three positions, either *R* or *S* product can be produced. However, the experiment showed that the *R* configuration was the major product (Dubey et al., 2016). For both the ω-1 and ω-2 positions, the MD simulations indicated that the pro-*R* C-H bond is closer to the ferryl-oxo moiety than the pro-*S* C-H bond, implying a preference of *R* product. In addition, the QM/MM calculations showed that for the ω-1 position, the barrier for the pro-*S* H abstraction is 6.3 kcal/mol higher than that for the pro-*R* H abstraction. In the case of the ω-3 position, even though the pro-*S* C-H bond is closer to the ferryl-oxo moiety than the pro-*R* C-H bond, the calculations using two different conformational basins (the major conformational basin and the minor conformational basin) showed that the barrier for the pro-*R* H-abstraction is lower than that for the pro-*S* H-abstraction (19.5 kcal/mol for TS_major−R_ vs. 26.2 kcal/mol for TS_major−S_, 19.2 kcal/mol for TS_minor−R_ vs. 21.7 kcal/mol for TS_minor−S_). These results suggested that despite that the initial proximity of the pro-*S* ω-3 C-H bond, the *R* product is still the dominant one.

### Non-heme-Dependent Enzymes

Non-heme-dependent enzymes are another superfamily of enzymes that participate in a large number of biological processes. Different from cytochrome P450, the active sites of these enzymes do not contain the porphyrin ligand. Much computational work has also been done on the selectivities of these enzymes (Karamzadeh et al., 2010; Suardíaz et al., 2013, 2014; Saura et al., 2014; Liao and Siegbahn, 2015; Christian et al., 2016; Roy and Kästner, 2017; Timmins et al., 2017; Wojdyla and Borowski, 2018), and some representative cases are shown here.

**Figure 7:**
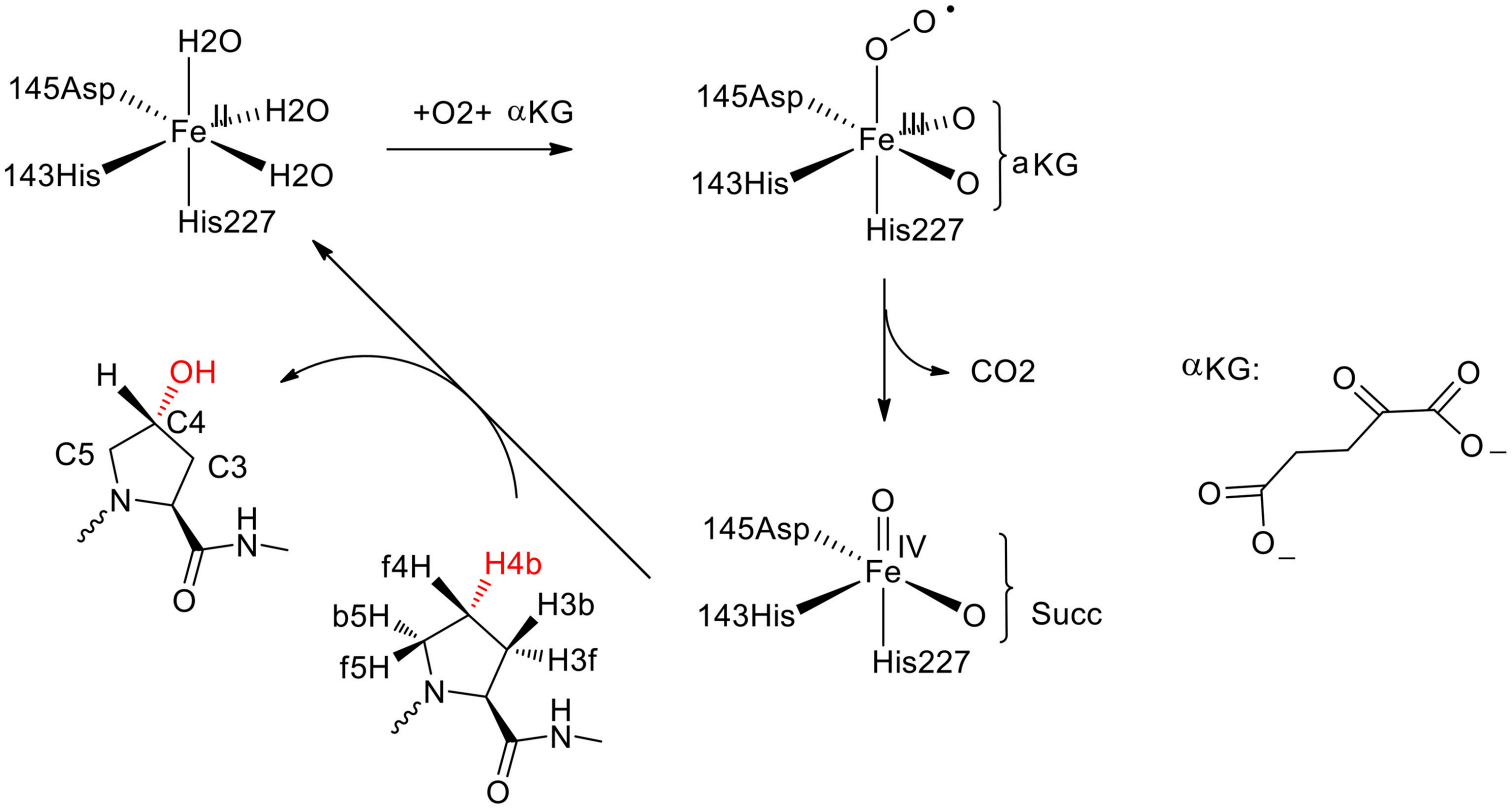
Suggested catalytic cycle of P4H (αKG represents α-ketoglutarate, and Succ represents succinate) (Karamzadeh et al., 2010; Timmins et al., 2017). Copyright (2017) American Chemical Society.

Prolyl-4-hydroxylase (P4H) is a non-heme iron hydroxylase that mediates the hydroxylation of a proline residue in a peptide chain to *R*-4-hydroxyproline with regioselectivity and stereoselectivity (Figure 7; Winter and Page, 2000). To rationalize the selectivities of this enzyme, the De Visser group has performed both the QM cluster and the QM/MM calculations (Karamzadeh et al., 2010; Timmins et al., 2017).

In the QM cluster calculations (Karamzadeh et al., 2010), quite a small model was used, consisting of the Fe with its first-shell ligands and the substrate. The calculations showed that the most thermochemical favorable hydroxylation takes place at the C5 position, which is inconsistent with the experiment observation (Winter and Page, 2000). The cluster model was then enlarged by including some important second-shell residues. It was found that the steric interactions imposed by Tyr140 and Trp243 increase the energy barriers for the hydroxylation at the C3 and C5 positions, leading to the preference of hydroxylation at the C4 position.

Recently, they performed molecular dynamics simulations and QM/MM calculations to find the origin of the regioselectivity and stereoselectivity in this enzyme (Timmins et al., 2017). Since their previous cluster calculations suggested that Tyr140 and Trp243 in the active site play an important role in the regioselectivity, both the wild-type and the mutant structures were taken into consideration. The rate-limiting step was found to be the hydrogen atom abstraction by the oxygen atom of the iron (IV)-oxo species. The hydroxylation at the C4 position (TS_HA_, _C4b_) has the lowest energy barrier at 20.7 kcal/mol, while they are 21.7, 50.0 kcal/mol for TS_HA_,_C5b_ and TS_HA_,_C3b_, respectively. For the Tyr140Phe mutant, the hydrogen bond between Tyr140 and the ferryl-oxo moiety vanishes, and the barrier for the hydroxylation at C4 position becomes over 50 kcal/mol, which suggested that the mutant is not active. When Tyr140 is replaced by a Gly group, the most favorable place becomes H4f and the C5 position is also feasible. It was demonstrated that the hydrogen bond between Tyr140 and the ferryl-oxo moiety dictates the regio- and stereoselectivity of the enzyme. For the Trp243Phe mutant, the QM/MM calculations showed that the most favorable hydroxylation takes place at the H5b position. While for the Trp243Gly mutant, the energy barriers for the hydroxylation at both C4 position and C5 position become over 30 kcal/mol. It was suggested that Trp243 along with Glu127 and Arg161 play an important role in orientating the substrate to a proper configuration for the hydroxylation.

**Figure 8:**
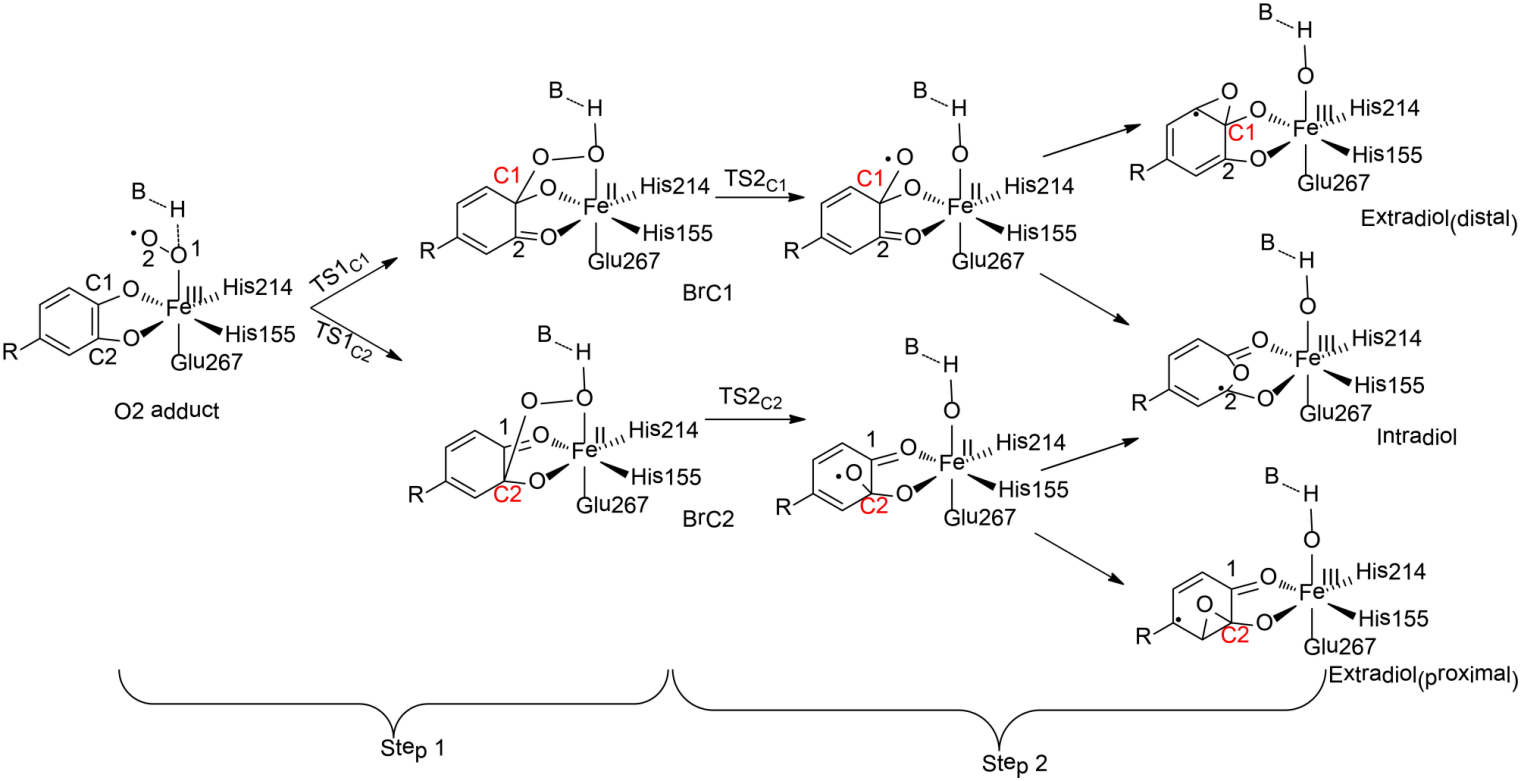
Reaction pathways considered for HCPD (Christian et al., 2016). Copyright (2016) American Chemical Society.

Homoprotocatechuate 2, 3-dioxygeanse (HCPD) is a non-heme iron extradiol dioxygenase that catalyzes C-C bond cleavage and ring opening of catecholates with high regioselectivity (Figure 8; Kovaleva and Lipscomb, 2007).

Christian and Ye have performed QM cluster calculations to elucidate the regiospecificity of HCPD (Christian et al., 2016). The calculations showed that the reaction started from the attack of the superoxide on the substrate with two possibilities (Step 1), namely at C1 and C2 positions, the barriers for which were calculated to be 31.3 kJ/mol and 28.3 kJ/mol, respectively. The resulting intermediate Br_C1_ is 28.4 kJ/mol higher in energy than Br_C2_.These results indicated that the selectivity for Step 1 is thermodynamically controlled. To further understand various factors that control the selectivity, different types of substrates have first been tested. When the native substrate Homoprotocatechuate (HPCA) was replaced by 2, 3-dihydroxybenzoate (2, 3-DHB), the energy difference between the attack at C1 and C2 is only 3.6 kJ/mol for the uncatalyzed reaction. Then, the influence of the first-shell coordination was investigated. The calculations showed that the energy of Br_C1_ is only 1.4 kJ/mol higher than Br_C2_ when using a model only containing the first-shell ligands. Thus, it is neither the substrate itself nor the first-shell coordination that controls the selectivity. Indeed, the selectivity of Step 1 is mainly controlled by a second residue Tyr257, which lowers the energies of the electron accepting orbitals (C2 = O2 π^*^ orbitals) and facilitates the C2-O2 bond formation. In addition, His200 was also found to facilitate the C2-O2 bond formation through geometric effect.

For Step 2, the influences of both the metal center and the coordination sphere were taken into consideration. Since the formation of the C1-O bond is unfavorable, the production of the distal extradiol can be ruled out. The calculations showed that three key second-shell residues (Tyr257, His200, and His248) dictate the selectivity of the proximal extradiol formation vs. the intradiol formation. In particular, Tyr257 has the greatest electronic effect, while His200 and His248 both have steric and electronic effects, and make the extradiol pathway more favorable.

**Figure 9:**
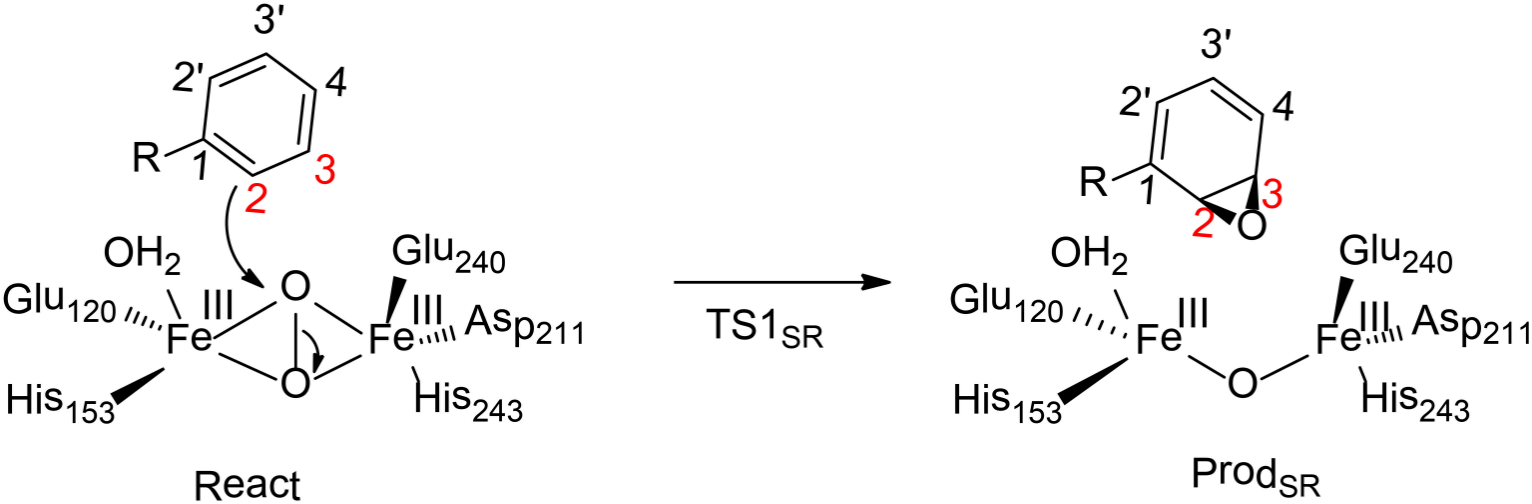
Reaction catalyzed by BoxB (Liao and Siegbahn, 2015).

Benzoyl-CoA epoxidase (BoxB) is a dinuclear iron enzyme that catalyzes the epoxidation reaction of the aromatic ring of benzoyl-CoA (Rather et al., 2011). In principle, there are two different types of aromatic oxidation, namely hydroxylation and epoxidation. For BoxB, only the epoxidation reaction was observed experimentally, even though it is thermodynamically less favorable compared to hydroxylation. In addition, the epoxidation can take place at three possible positions, namely 1, 2-position, 2, 3-position or 3, 4-position (Figure 9). However, only 2, 3-epoxide was obtained. Furthermore, the epoxide product has a configuration of (2S, 3R), suggesting a stereoselectivity (Rather et al., 2010).

To clarify the mechanism and the various selectivities of BoxB, Liao, and Siegbahn have performed density functional calculations on this enzyme with quite a large cluster model of 208 atoms (Liao and Siegbahn, 2015). The calculations suggested that during the binding of the dioxygen molecule, each ferrous ion delivers an electron to the dioxygen moiety to generate a superoxide bridging the two ferric ions in a side-on symmetric fashion. The two high-spin ferric ions interact in an antiferromagnetic fashion to form a broken-symmetry singlet species. From the reactant, the cleavage of the O-O bond turned out to lead to a simultaneous attack of one of the oxygen atoms on the substrate aromatic carbon. Interestingly, only epoxidation at the 2,3-position and 2′,3′-position can be located. The energy barriers for TS1_2S,3R_ and TS1_2R,3S_ are 17.6 and 20.4 kcal/mol, respectively. Based on the classical transition state theory, the energy difference of 2.8 kcal/mol corresponds to a selectivity of more than 99%:1%, which agrees with the experimental observation (Rather et al., 2010). In addition, a distortion/interaction analysis (Ess and Houk, 2008; Fernández and Bickelhaupt, 2014; Bickelhaupt and Houk, 2017) was used to understand the origin of selectivity. The calculations showed that the distortion energies are quite similar (24.3 kcal/mol for TS1_2S,3R_ and 24.8 kcal/mol for TS1_2R,3S_), while the interaction energy for TS1_2S,3R_ (18.3 kcal/mol) is somewhat smaller than TS1_2R, 3S_ (21.2 kcal/mol). Thus, the selectivity of BoxB was suggested to be mainly interaction-controlled.

To understand the chemoselectivity, the isomerization of epoxide to phenol was also taken into consideration. The C-O bond cleavage and deprotonation was found to proceed concertedly, associated with a barrier of 19.2 kcal/mol. This process was suggested to be slower than the release of the epoxide product from the enzyme active site.

Later, Rokob used the ONIOM (B3LYP/BP86/Amber) method to re-investigate this enzyme (Rokob, 2016). Four different pathways were considered for the aromatic ring oxidation, namely an electrophilic attached by a bis(μ-oxo)-diiron(IV) species, electrophilic attack via the σ^*^ orbital of a μ-η^2^:η^2^-peroxo-diiron(III) intermediate, radical attach via the π^*^-orbital of a superoxo-diiron(II,III) species, and radical attach of a partially quenched bis(μ-oxo)-diiron(IV) intermediate (Rokob, 2016). Importantly, the most favorable pathway (barrier of 20.9 kcal/mol) was found to be very similar as those obtained by Liao and Siegbahn (Liao and Siegbahn, 2015).

### Cobalt-Dependent Enzymes

PceA is a cobalamin-dependent reductive dehalogenase that catalyzes the dechlorination of perchloroethylene (PCE) to trichloroethylene (TCE), to *cis*-dichloroethylene (*cis*-DCE), and further to monochloroethylene (MCE) (Bommer et al., 2014). Three possible products can be envisioned for the dechlorination of TCE, namely, 1,1-DCE, *cis*-DCE, and *trans*-DCE. However, the *cis*-DCE was found to be the sole product, implying that PceA is regioselective.

**Figure 10:**
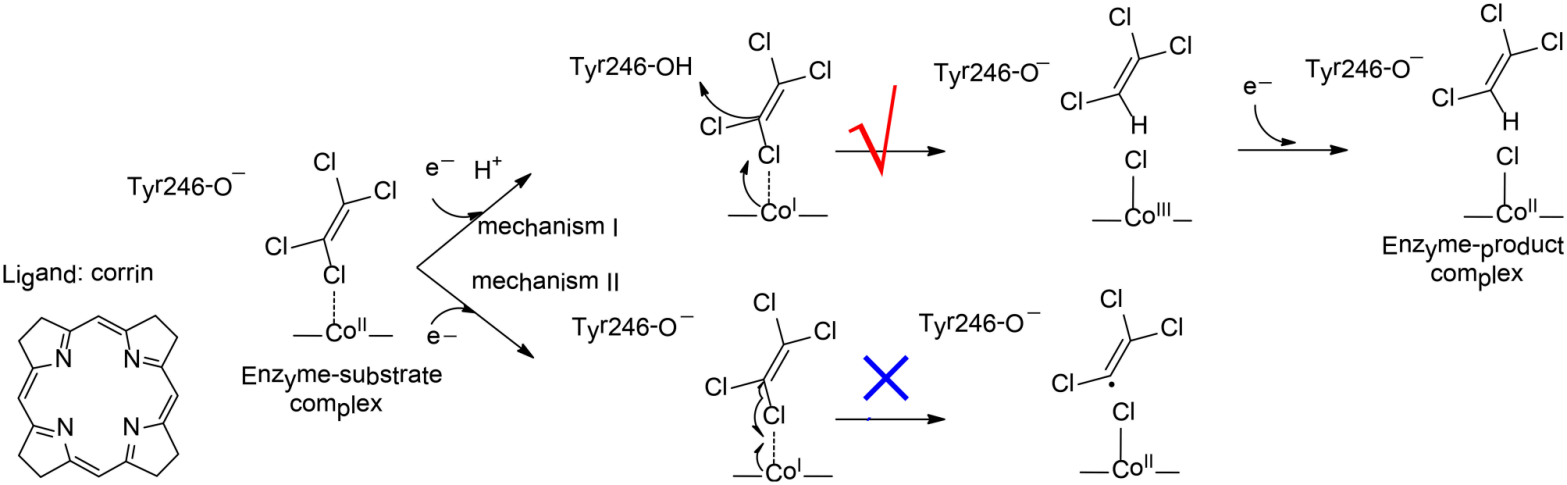
Reaction pathways considered for PceA (Liao et al., 2016). Copyright (2016) John Wiley and Sons.

To elucidate the reaction mechanism and regioselectivity of PceA, Liao et al. performed density functional calculations on this enzyme with quite a large model of 215 atoms (Liao et al., 2016). The dechlorination of PCE was first considered, and two different pathways have been analyzed (Figure 10). For pathway I, one electron reduction of Co^II^ to generate Co^I^ is coupled with the protonation of the second-shell anionic Tyr246 residue. This proton-coupled electron transfer step has a potential of −0.91 V. Then, a heterolytic C-Cl bond cleavage takes place, in concomitant with the proton transfer from Tyr246 to C1. During the reaction, the Co^I^ delivers two electrons to the Cl^+^, leading to the formation of a Co^III^-chloride complex. This step is rate-limiting, with a total barrier of 12.5 kcal/mol. Finally, the other one electron transfer to the metal center results in the formation of the Co^II^ product complex, and the whole reaction is exergonic by 36.0 kcal/mol. For pathway II, Tyr246 keeps anionic during the first one electron reduction. As a consequence, the following C-Cl bond cleavage is a homolytic process, and a Co^II^-chloride substrate radical complex is produced. This step is associated with a barrier of 15.1 kcal/mol, which is slightly higher than that for pathway I. Thus, mechanism I is kinetically more favorable, which is in line with the suggested mechanism for the other reductive dehalogenase NpRdhA (Liao et al., 2015).

To rationalize the regioselectivity in the dechlorination of TCE, all six possible substrate binding modes inside the active have been considered. There are two substrate orientations for the formation of each product, *cis*-DCE, *trans*-DCE, and 1,1-DCE. The lowest energy barriers for the formation of *cis*-DCE, *trans*-DCE, and 1,1-DCE were calculated to be 13.8, 17.6, 18.4 kcal/mol, respectively. It was suggested that the amide group of cobalamin and other important second-shell residues form a pocket that favors the formation of the *cis*-DCE product but imposes larger steric repulsion in the formation of the other products.

The dechlorinations of *cis*-DCE and MCE have also been considered, and the corresponding barriers were calculated to be 20.8, 27.6 kcal/mol, respectively. The calculations showed that the energies of the HOMOs for PCE, TCE, *cis*-DCE, and MCE were −7.12, −7.11, −7.07, −7.15 eV, respectively, while the energies of the LUMOs were −1.11, −0.82, −0.42, −0.07 eV, respectively. During the dechlorination, an electron is transferred from Co^I^ to the LUMO of the substrate. Therefore, the substrate with lower LUMO was suggested to be more reactive, and with lower barrier.

Recently, Ji et al. investigated the cobalamin-mediated reductive dehalogenation reaction by DFT calculations (Ji et al., 2017). Both the inner-sphere and the out-sphere pathways have been considered. The comparison of the calculated kinetic isotope effect and the experimental kinetic isotope effect was used as a probe for diagnosing the reaction mechanism. The reaction in water solution was suggested to proceed via the formation of an organometallic intermediate with a Co-C bond (Ji et al., 2017). This is different from the case in enzyme, in which a second-shell residue was suggested to protonate the substrate during the reductive dehalogenation reaction (Liao et al., 2016).

Johannissen et al. have performed molecular docking and DFT calculations to compare the interactions between the substrate and the cobalamin in both NpRdhA and PceA (Johannissen et al., 2017). A [Co•••X•••R] adduct was suggested to be formed at the Co^I^ state, which weakens the substrate carbon-halide bond. This is evidenced by the elongation of the carbon-halide bond during the reduction of Co^II^ to Co^I^, and similar results have been found by Liao et al. (2016).

### Nickel-Dependent Enzymes

**Figure 11:**
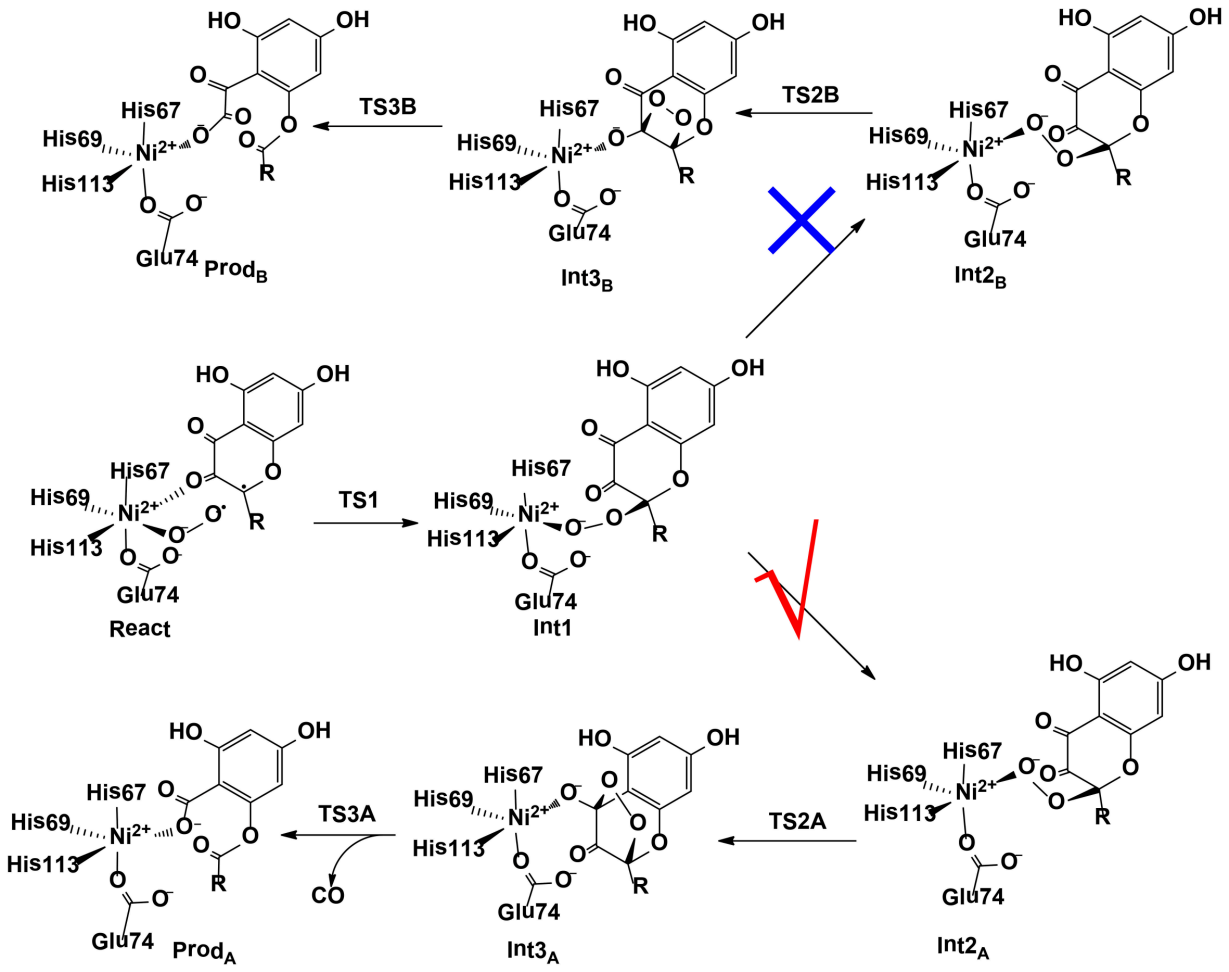
Two possible pathways considered for Ni-QueD (Wang et al., 2018). Copyright (2018) Royal Society of Chemistry.

Wang et al. (2018) have investigated the mechanism and chemoselectivity of a nickel-dependent quercetin 2,4-dioxygenase (Ni-QueD) (Jeoung et al., 2016) by performing QM/MM calculations at the B3LYP-D3/def2-TZVPP:Charmm level (Figure 11). In the dioxygenation of quercetin, two possible pathways can be envisioned, the 2,4-dioxygenolytic cleavage to harvest 2-protocatechuoylphloroglucinol carboxylic acid and carbon monoxide, and the 2,3-dioxygenolytic cleavage to produce α-keto acid. The formal pathway was observed exclusively by experiment, suggesting a chemoselectivity of this enzyme.

In their QM/MM calculations, the critical first-shell ligand Glu74 has been considered to be both in the neutral and in the ionized form. It was found that Glu74 must be deprotonated to favor the 2,4-dioxygenolytic pathway and to rationalize the chemoselectivity. The binding of a dioxygen molecule to the Ni^II^ ion results in the formation of an open-shell broken-symmetry singlet species, in which a triplet Ni^II^ is antiferromagnetically-coupled with the triplet dioxygen moiety, with partial electron transfer from the anionic quercetin substrate to the dioxygen moiety. However, the following reaction takes place preferentially in the triplet state, and a spin-crossing has to take place during the first C-O bond formation, which leads to the generation of a Ni^II^-peroxide intermediate. Prior to the second C-O bond formation, a conformation change take place, which is required for the second C-O bond formation. Subsequently, the peroxide could attack either C3 or C4. The barrier for the attack on C4 is 3.6 kcal/mol lower than that on C3. The attack on C4 results in the formation of a pentabasic cyclic intermediate, from which the ring opening takes place with the release of a carbon monoxide molecule. This step was calculated to be rate-limiting, with a barrier of 17.4 kcal/mol. Alternatively, the opening of the four-membered ring after the attack on C3 is associated with a barrier of 30.6 kcal/mol. The calculations showed that the 2,4-dioxygenolytic pathway is much more favored than the 2,3-dioxygenolytic pathway. In addition, QM gas phase calculations and QM/MM calculations using mechanic embedding scheme also favor the 2,4-dioxygenolytic pathway significantly. The calculated barrier of 17.4 kcal/mol agrees quite well with the experimental kinetic constant of 40.1 s^−1^ (Merkens et al., 2008), which corresponds to barrier of about 15 kcal/mol.

Liu and co-workers have also investigated the reaction mechanism of this enzyme using the QM/MM method. However, only the 2,4-dioxygenolytic pathway was considered, and a similar mechanism was suggested (Li et al., 2018).

### Zinc-Dependent Enzymes

Moa and Himo have used the quantum chemical cluster approach to investigate the stereoselectivity of the zinc-dependent secondary alcohol dehydrogenase (Moa and Himo, 2017). In order to rationalize the opposite enantioselectivity in the dehydrogenation of 2-butanol (*R*-selective) and 3-hexanol (*S*-selective), an active site model of more than 300 atoms were designed from the crystal structure.

The calculations support the general mechanism as proposed for other alcohol dehydrogenase (Cui et al., 2002), in which a proton is first transferred from the substrate alcohol to a neutral histidine on the enzyme surface, followed by a hydride transfer from the substrate alkoxide to the NADP^+^ cofactor. The zinc ion functions as a Lewis acid to stabilize the alkoxide intermediate. For 2-butanol, two different substrate orientations have been found for both the *(R)*- and *(S)*-enantiomers. Interestingly, the orientation with lower energy is always non-productive. In addition, the small cavity in the active site prefers to bind the ethyl group for both 2-butanol and 3-hexanol. This is quite unexpected for 2-butanol, for which the large cavity prefers to bind the smaller methyl group. The energy decomposition analysis (Kitaura and Morokuma, 1976) showed that larger attractive dispersion interaction is presented when the ethyl group is in the small cavity. This also explains the preference of the *(R)*-TS compared with the *(S)*-TS (energy difference of 1.3 kcal/mol) for 2-butanol. Similar analysis have been performed for 3-hexanol, for which the *(S)*-TS is now preferred by 4.2 kcal/mol compared with the *(R)*-TS. Compared with the experimental data, the barrier difference between the *(R)*- and *(S)*-enantiomers were slightly overestimated, which was suggested to originate from the constrain of the model during the geometry optimization. More flexibility of the binding pockets by using even larger models is needed to rationalize the enantioselectivity of substrates with even larger substituents.

It should be pointed out that the reproduction of stereoselectivity in enzymes has been considered to be very challenging, as it requires accurate calculations of very small energy differences between different transition states. However, the Himo group showed that the quantum chemical cluster methodology is capable of solving this kind of important question, evidenced by four additional examples, namely limonene epoxide hydrolase (Lind and Himo, 2013), arylmalonate decarboxylase (Lind and Himo, 2014), soluble epoxide hydrolase (Lind and Himo, 2016), and phenolic acid decarboxylase (Sheng and Himo, 2017).

### Molybdenum-Dependent Enzymes

**Figure 12:**
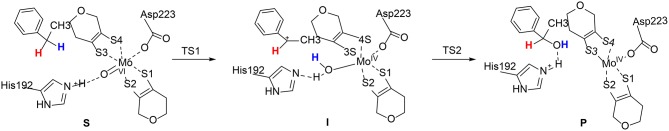
Suggested mechanism for molybdenum-dependent ethylbenzene dehydrogenase (Szaleniec et al., 2014). Copyright© 2014 Elsevier Inc.

Szaleniec et al. reported a QM/MM studies on the enantioselectivity of the molybdenum-dependent ethylbenzene dehydrogenase (Szaleniec et al., 2014; Figure 12). A two layered ONIOM method (B3LYP/Lacv3p^**^: Amber) was used for the calculations. A small QM region of 52 atoms (QM1) was used for the initial calculations, followed by calculations using a large QM region of 168 atoms (QM2). The oxidation of the ethylbenzene substrate was suggested to be divided into two phases, namely C-H activation and OH-rebound. The steric effects imposed by the enzyme active site enforce an almost planar conformation of ethylbenzene, with the pro(*S*) H-atom pointing to the Mo(VI) = O moiety. The first C-H activation step turns out to be a proton-coupled two-electron transfer process. A radical substrate is formed at the transition state (TS1) and it becomes a carbon cation at the intermediate. During the reaction, the second-shell residue His192 delivers a proton to the Mo(VI) = O moiety. In the second step, the Mo(IV)-bound water molecule performs a nucleophilic attack on the carbon cation intermediate (TS2), coupled with a proton transfer from this water to His192. The first step was calculated to be rate-limiting, with a barrier of 84.4 kJ/mol using QM2, and the second OH-rebound step was found to be barrierless.

On the basis of the proposed mechanism, the enantioselectivity was then analyzed by comparing the relative energies of all stationary points for the formation of both (*S*)- and (*R*)-phenylethanol. The energy difference between TS1_pro(S)_ and TS1_pro(R)_ was calculated to be 17.2 kJ/mol favoring the (*S*)-pathway, while the energy difference decreases to 8.6 kJ/mol for the second transition state. The interaction energies between the substrate and the nearby active site residues were used to analyze the source of the enantioselectivity. The corresponding values were calculated to be −63.7 and −48.6 kJ/mol for TS1_pro(S)_ and TS1_pro(R)_, respectively.

### Tungsten-Dependent Enzymes

The mechanism and selectivity of three different tungstoenzymes have been subjected to QM and QM/MM calculations, namely, acetylene hydratase (Liao et al., 2010; Liao and Himo, 2011), formaldehyde oxidoreductase (Liao et al., 2011b; Liao, 2013) and benzoyl CoA reductase (Culka et al., 2017; Qian and Liao, 2018).

**Figure 13:**
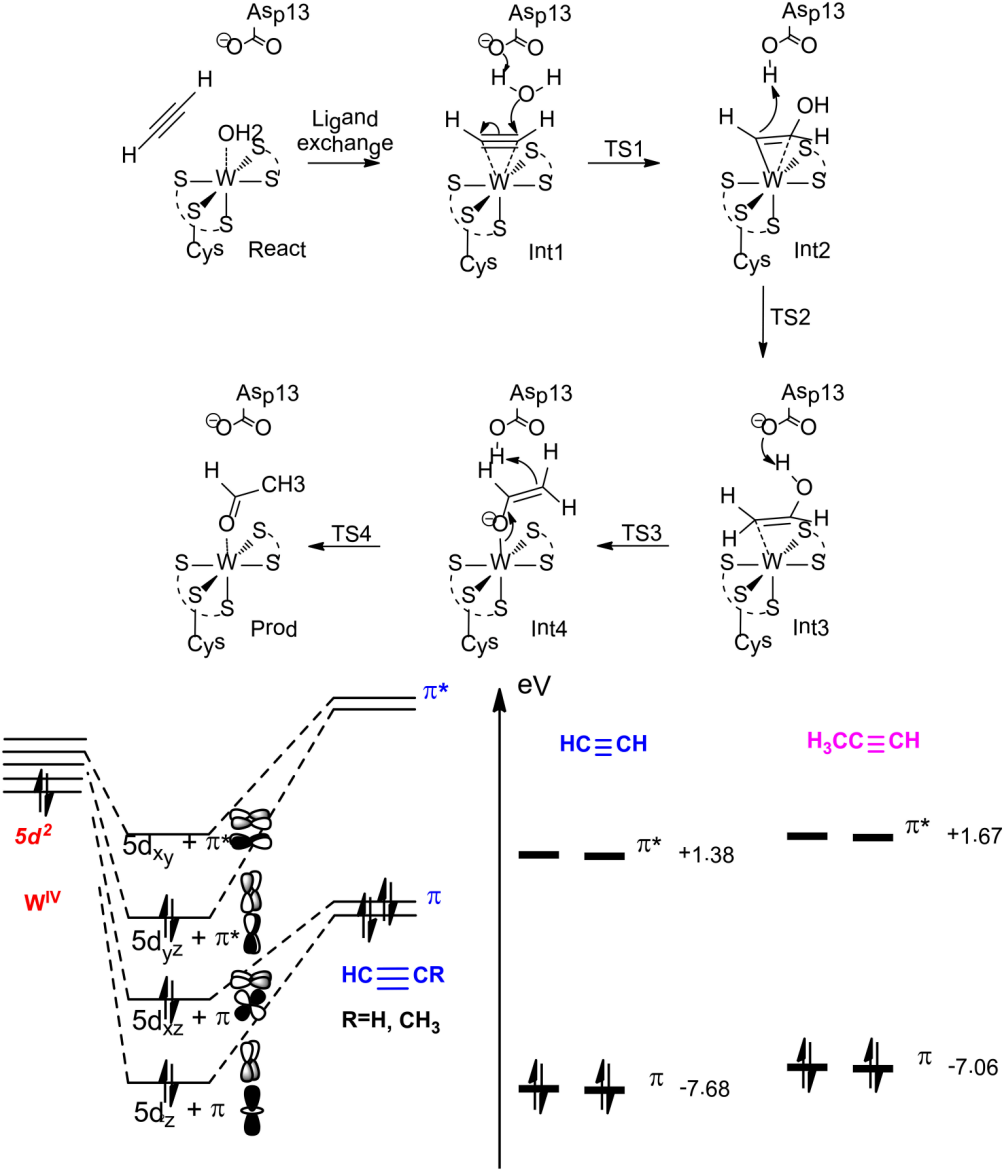
Suggested mechanism for acetylene hydratase (Top), orbital interaction diagram in Int1 (left down), and HOMO-LUMO energy level for acetylene and propyne (right down) (Liao and Himo, 2011). Copyright (2011) American Chemical Society.

On the basis of QM calculations with a model of 116 atoms, Liao et al. (2010) suggested a first-shell mechanism that gave reasonable barrier (Figure 13). The reaction starts with a ligand exchange of a W(IV)-bound water molecule by the acetylene substrate. In the next step, the liberated water molecule, which gets deprotonated by the second-shell anionic Asp13 residue, performs a nucleophilic attack on the W (IV)-bound acetylene. This leads to the formation of a W(IV)-vinyl anion intermediate, which undergoes protonation by the Asp13 to produce a W(IV)-vinyl alcohol complex. This step was calculated to be rate-limiting with a barrier of 23.0 kcal/mol at the B3LYP/ LANL2TZ(f)-6-311+G(2d,2p) level. The isomerization of the vinyl alcohol to acetaldehyde catalyzed by the enzyme by two sequential proton transfer steps was found to be quite facile.

This new mechanism was then used to rationalize the chemoselectivity of this enzyme (Liao and Himo, 2011), which does not catalyze the hydration of either propyne or ethylene. The calculations showed that the ligand exchange of water by propyne is more exothermic than that by acetylene, and the following hydration has a much higher barrier for propyne, being over 30 kcal/mol. This explains that propyne is a competitive inhibitor as observed from experiment. The different reactivity of acetylene vs. propyne has been explained by analyzing the orbital interactions between the W(IV) ion and the substrate. W(IV) has an electronic configuration of 5d^2^, the two degenerate π orbitals of the substrate (acetylene and propyne) interact with two empty 5d orbitals of W(IV), and the occupied 5d orbital of W(IV) interacts with one of the two unoccupied π^*^ orbitals via back-donation. The other unoccupied π^*^ orbital of the substrate interacts with one of the remaining unoccupied 5d orbitals of W(IV) to generate a δ-like orbital, which facilitates the electron transfer to the empty π^*^ orbital during the following nucleophilic attack of the water molecule on the substrate. Upon methyl substitution, the HOMO energy raises by 0.6 eV from acetylene to propyne, and this results in better π donation and larger binding energy for propyne. In addition, the LUMO energy also raises by 0.3 eV from acetylene to propyne, and this makes the electron transfer to the δ-like orbital more difficult during the nucleophilic attack. Steric repulsion has been suggested to be another plausible reason for the different reactivity of acetylene and propyne.

For the hydration of ethylene, the ligand exchange of water by ethylene becomes slightly endothermic, and the barrier for the following water attack has a barrier of more than 30 kcal/mol. The orbital interaction analysis showed that the two electron donor character of ethylene makes its π donation much weaker than that of acetylene. In addition, the back-donation of the occupied 5d orbital of W(IV) to the only unoccupied π^*^ orbital of ethylene results in the involvement of an even higher π^*^-like orbital to accept the electron during the following nucleophilic attack, which is much less favorable.

**Figure 14:**
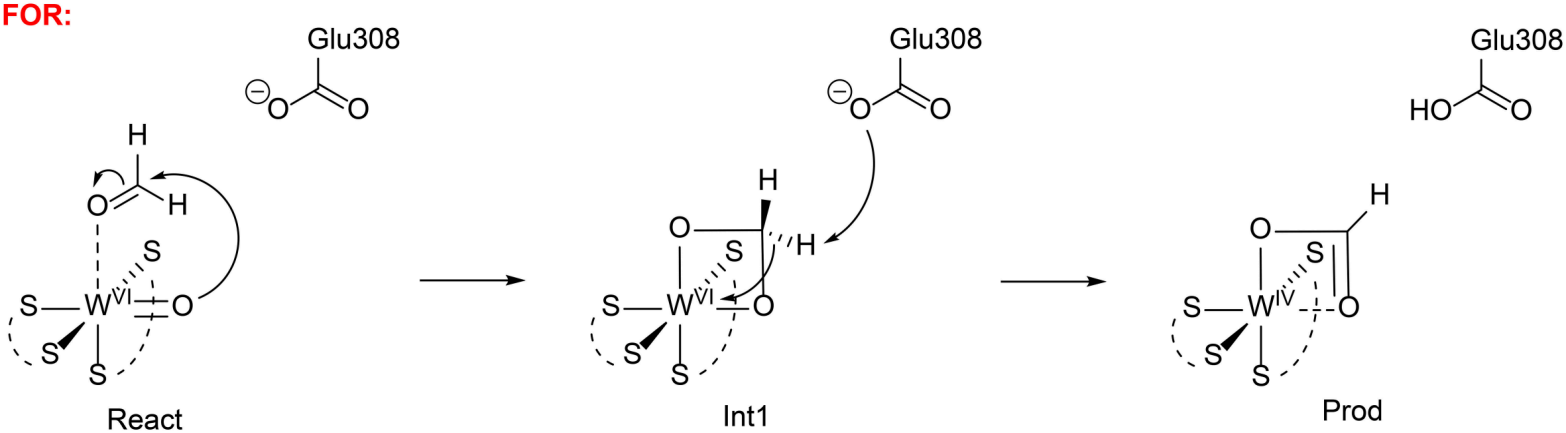
Suggested mechanisms for FOR (Liao et al., 2011b). Copyright (2011) Elsevier Inc.

Liao et al. has also investigated the mechanism of formaldehyde oxidoreductase (FOR) and the W vs. Mo selectivity for this enzyme (Liao et al., 2011b). QM calculations showed that this enzyme uses a W(VI) = O as the key oxidant and the formaldehyde coordinates to W(VI) directly via its oxygen atom. The W(VI) = O moiety then performs a nucleophilic attack on the formaldehyde carbon, generating a tetrahedral intermediate (Figure 14). Subsequently, a second-shell anionic residue Glu308 abstracts a proton from the tetrahedral intermediate, coupled with two-electron transfer from the intermediate to W(VI), which becomes reduced to W(IV). Other possible pathways have also been considered but were found to have much higher barriers. The suggested mechanism was then used to explain why the molybdenum substituted enzyme is not active, even though some other enzymes are able to use both molybdenum and tungsten for catalysis (Liao, 2013). The whole catalytic cycle including the formation of the active oxidant M(VI) = O (M = Mo and W) and the oxidation of formaldehyde were considered. The resting state of the enzyme is M(IV)-OH_2_ and its oxidation to M(VI) = O involves two sequential proton-coupled electron transfer steps. For W-FOR, the energetics for the two oxidation steps can be estimated from the experimental redox potentials of the external electron acceptor [Fe_4_S_4_]^2+/+^ (−350 mV) and the W(IV)/W(V) and W(V)/W(VI) couples. The formation of the active reactant complex is exothermic by 1.7 kcal/mol. For Mo-FOR, the relative redox potentials, which can be obtained with very good accuracy as the metal has the same environments before and after the oxidation, were calculated and used to set up the energetics. The formation of the active reactant complex now becomes endothermic by 11.7 kcal/mol. This large difference originates from the different redox potentials of W and Mo, which have also been observed experimentally for the redox potentials of pairs of molybdenum and tungsten complexes. The oxidation of formaldehyde by Mo-FOR proceeds via a similar pathway as that by W-FOR. However, due to the energetic penalty for the formation of the active Mo(VI) = O, the total barrier increases to 28.2 kcal/mol (17.6 kcal/mol for W-FOR).

**Figure 15:**
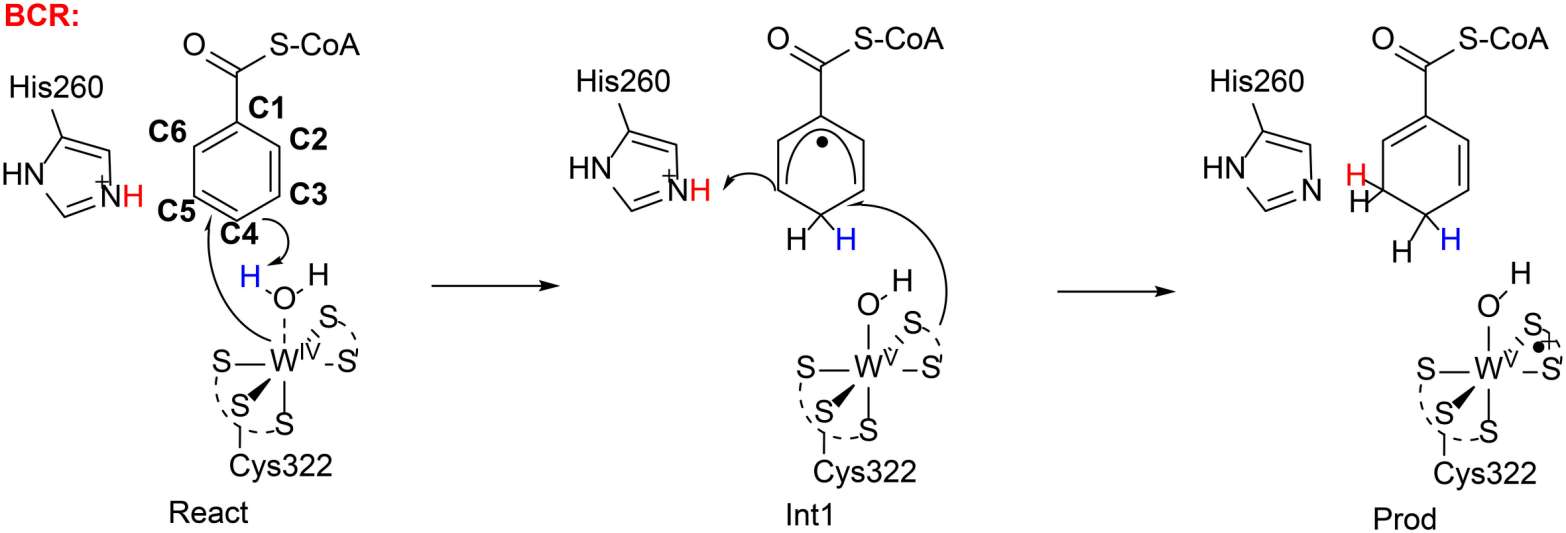
Suggested mechanisms for BCR (Qian and Liao, 2018). Copyright (2018) American Chemical Society.

Very recently, Qian and Liao performed QM/MM calculations (B3LYP-D3/def2-TZVPP:Charmm) to rationalize the regioselectivity of the benzoyl CoA reductase (Qian and Liao, 2018). They found a similar mechanism (Figure 15) as suggested by Culka et al. (2017) on the basis of QM/MM calculations. In the reactant complex, a water molecule is coordinated to the W(IV) ion. The reduction of the benzene ring proceeds via two sequential steps. First, the W(IV)-bound water molecule delivers a proton to the *para*-carbon (C4) of the benzene ring, in concomitant with an electron transfer from the W(IV) center to the substrate. Consequently, a W(V)-OH cyclohexadienyl radical intermediate is generated. The second step involves a proton transfer from a second-shell residue His260 to the *meta*-carbon (C3) of the cyclohexadienyl radical and an electron transfer from the pyranopterin cofactor to the substrate. The first step was calculated to be rate-limiting, with a barrier of 23.2 kcal/mol in the broken-symmetry singlet state.

The reduction of the aromatic ring at other positions was then considered. Geometry constrains dictates that only the two other metal-carbons (C3 and C5) could accept the proton from the water molecule during the first reduction step. The barriers for these two pathways were found to be over 8 kcal/mol higher than that for the proton transfer to C4. The preference for the *para* attack was partially explained by the extra spin delocalization to the adjacent carbonyl group in the cyclohexadienyl radical intermediate, and the extra spin delocalization is absent for the *meta* attack. In addition, the substrate orientation also favors the attack on C4. For the reduction at both 2,3 and 5,6-positions, the second step turns out to be rate-limiting, with barriers of more than 30 kcal/mol. The calculations thus reproduce the regioselectivity.

They have also investigated the substitution of tungsten by molybdenum to predict if the Mo-BCR is active or not. The reaction mechanism is generally the same, however, the barrier increases to 31.1 kcal/mol in the open-shell singlet for Mo-BCR. In addition, the reduction becomes endothermic by more than 15 kcal/mol. The main reason was suggested to originate from the different redox potentials of the M(VI)/M(V) and M(V)/M(IV) couples (M = Mo and W), and tungsten has more negative potentials than molybdenum, as found previously from both experimental and theoretical studies.

## Summary and Outlook

In this review, we have presented the progress of the computational modeling of selectivities in metalloenzymes. Both the quantum chemical cluster and the QM/MM approaches have shown to be successfully applied in the rationalization of selectivities and identification of factors that control the selectivity.

One of the most important questions in these enzymes, is the origin of the various selectivities, namely chemoselectivity, regioselectivity, stereoselectivity, metal oxidation preference (Fe^2+^ vs. Fe^3+^), and metal selectivity (W vs. Mo). Mn, Fe, and Ni are typically used in the activation of dioxygen for oxidative transformations. The selectivity is typically controlled by the protein environment, and a proper description of the substrate surroundings is crucial for the rationalization of the selectivity. In Fe-dependent aldoxime dehydratase, the redox nature of the mechanism dictates the use of a lower oxidation state Fe^2+^ for electron delivering, rather than the more Lewis acidic Fe^3+^. In the case of Mn-dependent FosA and Zn-dependent secondary alcohol dehydrogenase, both metal ions mainly function as a Lewis acid to stabilize the oxy anion during the reaction. Co is used for reductive dehalogenation, and the regioselectivity is controlled by the active site pocket. The stereoselectivity in the Mo-dependent ethylbenzene dehydrogenase is also controlled by the active site. For tungsten-dependent enzymes, the tungsten ion in acetylene hydratase enables orbital interactions with the substrate, which illuminates the chemoselectivity. While for both formaldehyde oxidoreductase and benzoyl CoA reductase, the different oxidation potential for the W^6+^/W^4+^ couple and the Mo^6+^/Mo^4+^ couple, explains the metal preference in these two specific examples.

There are a number of challenging issues that need to be considered during the modeling of selectivities. First, a correction mechanism that fits all available experimental observations has to be suggested first. This is a known challenge for reactions where electrons and protons are penetrated or liberated, as the methods currently available are not accurate enough to determine the absolute p*K*_a_s and redox potentials. Second, the reproduction of selectivity, especially stereoselectivity, requires an accuracy of < 1 kcal/mol. For many cases, error cancellation may be present, which makes rationalization possible. The standard DFT/MM method is a suitable choice for such applications. In other very few cases, one may need to push the accuracy limit by minimizing all possible errors and considering various issues, such as the effect of entropy and conformation, the use of high-level *ab* initio methods, the use of proper and larger QM region, the use of better force field, et al. in the QM/MM calculations. In practice, this may be very difficult to achieve due to the tradeoff between accuracy and speed. The most challenging issue is to make a prediction that can be confirmed by an experiment. This is extremely important in the field of directed evolution, which is capable of manipulating the selectivity using active site mutations. This area is likely where extraordinary efforts will be dedicated in the future.

## Author Contributions

All authors listed have made a substantial, direct and intellectual contribution to the work, and approved it for publication.

### Conflict of Interest Statement

The authors declare that the research was conducted in the absence of any commercial or financial relationships that could be construed as a potential conflict of interest.
